# Bone Aging and Glycative Stress: Convergent and Divergent Mechanisms Driving Skeletal Deterioration

**DOI:** 10.3390/cells15181712

**Published:** 2026-09-20

**Authors:** Salvador Peñarrubia, Eduardo Martín-Guerrero, Arancha R. Gortázar, Juan A. Ardura

**Affiliations:** 1 Department of Orthopaedics, Hospital Universitario Fundación Jiménez Díaz, 28040 Madrid, Spain; 2 Bone Physiopathology Laboratory, Instituto de Medicina Molecular Aplicada (IMMA), School of Medicine, Universidad San Pablo-CEU, CEU Universities, Urbanización Montepríncipe, 28925 Alcorcón, Spain; 3 Department of Basic Medical Sciences, School of Medicine, Universidad San Pablo-CEU, CEU Universities, Campus Monteprincipe, 28925 Alcorcón, Spain

**Keywords:** bone, aging, glycative stress, AGEs, osteoporosis, signaling pathways

## Abstract

Aging and glycative stress are major, interrelated drivers of skeletal fragility, yet the extent to which they act through shared versus distinct biological pathways remains poorly defined, limiting integrated therapeutic strategies. This review compares the convergent and divergent mechanisms by which aging and glycative stress affect osteocytes, osteoblasts, and osteoclasts, extracellular matrix properties, and bone mechanotransduction. Both conditions converge on oxidative stress, mitochondrial dysfunction, chronic low-grade inflammation, cellular senescence, impaired autophagy, NLRP3 inflammasome activation, and ferroptosis, ultimately reducing osteocyte viability and disrupting RANKL/OPG-mediated remodeling. They diverge in their primary drivers: Aging is characterized by hormonal decline, stem-cell exhaustion, and progressive loss of bone mass and microarchitecture, whereas glycative stress acts through AGE–RAGE signaling and collagen cross-linking, compromising bone quality and mechanosensitivity while often preserving bone mineral density, explaining the disproportionate fracture risk seen in diabetes. Since current anabolic and anti-resorptive therapies do not specifically target AGE-related pathways, combined strategies incorporating senolytic, antiglycative, and mechanoprotective approaches—alongside lifestyle interventions—may be needed to more effectively reduce fracture risk in aged and diabetic populations.

## 1. Introduction to Aging and Glycative Stress in Bone

Bone is a highly dynamic and mechanically responsive tissue that continuously adapts its structure and function to meet both mechanical and metabolic demands. This adaptive capacity, known as mechanoadaptation, enables the skeleton to sense and respond to external mechanical forces [1]. The homeostasis of the two major compartments of bone—cortical (compact) and trabecular (cancellous)—is maintained through processes such as remodeling [2]. Bone remodeling is a lifelong process in which old or damaged bone is removed by osteoclast-mediated resorption and subsequently replaced by osteoblast-derived bone formation, followed by matrix mineralization [3]. Osteocytes, the most abundant bone cell type, play a central role in coordinating this process through their ability to sense mechanical and hormonal cues, regulate mineralization, and secrete paracrine and endocrine factors that influence cells within the bone and bone marrow microenvironment [4].

Aging profoundly affects skeletal health and is one of the major risk factors for common bone disorders such as osteoporosis and fragility fractures. During aging, the balance of bone remodeling progressively shifts toward increased bone resorption and reduced bone formation, resulting in net bone loss [5]. These alterations lead to characteristic structural changes, including reductions in trabecular thickness and trabecular bone volume, as well as increased cortical porosity, which collectively compromise bone mass and strength and fracture resistance [6,7].

Aging-related bone deterioration arises from the interaction of intrinsic and extrinsic factors, including genetic predisposition, endocrine changes, oxidative stress, glycative stress, and chronic low-grade inflammation. In particular, the age-associated decline in sex steroid hormones, accumulation of reactive oxygen species (ROS), and increased production of pro-inflammatory cytokines contribute to skeletal fragility by disrupting bone cellular function and promoting osteoclastogenesis [8]. A wide body of research has identified a series of conserved biological mechanisms, collectively known as the hallmarks of aging, which contribute to age-related functional decline in different organs, including bone. López-Otín and colleagues first proposed nine hallmarks of aging in 2013, including genomic instability, telomere attrition, epigenetic alterations, loss of proteostasis, deregulated nutrient sensing, mitochondrial dysfunction, cellular senescence, stem cell exhaustion, and altered intercellular communication. More recently, this framework has been expanded to incorporate disabled macroautophagy, dysbiosis, and chronic inflammation (inflammaging). These hallmarks share several common features: They emerge progressively during aging, contribute causally to age-related dysfunction, and represent potential targets for therapeutic intervention [9]. Age-related alterations affect the major bone cell populations involved in skeletal homeostasis, namely osteocytes, osteoblasts and osteoclasts, as discussed in the following sections.

In parallel to aging hallmarks, growing evidence supports glycative stress as an important contributor to biological and skeletal aging [10]. Glycative stress is defined as the cellular state in which advanced glycation end-products (AGEs), advanced glycoxidation end-products (AGOEs) and their reactive dicarbonyl precursors, such as methylglyoxal (MGO), accumulate at a pathological rate [11]. This imbalance arises from two conditions: one, from an increase in their formation, driven by hyperglycaemia, glycaemic variability or high-glycaemic-index diets, but also by aging-dependent or independent oxidative stress, mitochondrial dysfunction, inflammation, smoking, impaired renal clearance… Second, from the failure of the cellular systems that normally detoxify them, namely the glyoxalase pathway, DJ-1/Park7, the ubiquitin-proteasome system (UPS) and autophagy, a self-degradation and recycling of cellular components process in cells. When these systems are overwhelmed, glycative modifications accumulate in extracellular matrix proteins and in intracellular components, altering cell function and activating pro-inflammatory, pro-oxidative and pro-apoptotic signaling pathways [12,13,14,15]. AGEs also favor mitochondrial dysfunction, cellular senescence, altered intercellular communication and AGE/AGOE buildup, creating a self-perpetuating cycle of glycative damage [16,17]. Thus, although classically associated with diabetes mellitus and chronic hyperglycemia, glycative stress is also a prominent feature of numerous pathological conditions including aging [10]. Consequently, glycative stress may be viewed both as a downstream consequence of aging-related metabolic alterations and as an independent driver of tissue degeneration [9,10]. The actions of glycative stress on osteocytes, osteoblasts and osteoclasts are discussed in the following sections.

**Figure 1:**
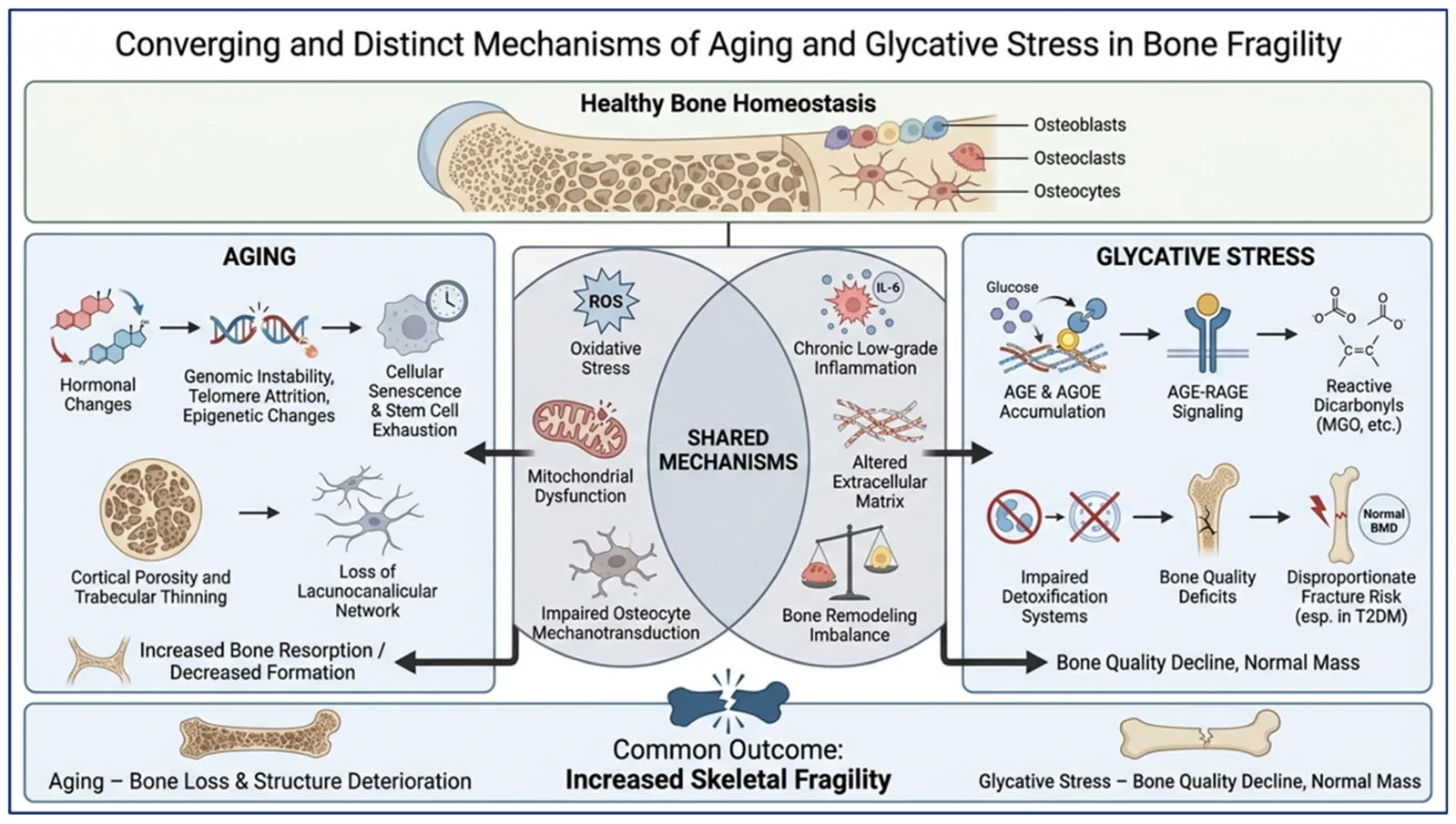
Overview of convergent and divergent mechanisms linking aging and glycative stress to skeletal fragility. Schematic overview comparing the effects of aging and glycative stress on bone health. **Top**: A longitudinal section of healthy bone illustrating homeostatic populations of osteoblasts, osteoclasts and osteocytes lining and embedded within the bone matrix. **Left panel** (“Aging”): Hormonal changes initiate genomic instability, telomere attrition and epigenetic alterations that drive cellular senescence and stem-cell exhaustion; concurrently, cortical porosity and trabecular thinning lead to loss of the lacunocanalicular network, together producing increased bone resorption and decreased bone formation. **Right panel** (“Glycative Stress”): Glucose reacts to form advanced glycation and glycoxidation end-products (AGEs/AGOEs) that engage AGE–RAGE signaling and generate reactive dicarbonyl intermediates such as methylglyoxal (MGO); impaired cellular detoxification systems produce bone quality deficits that translate into a disproportionately high fracture risk despite normal bone mineral density (BMD), particularly in type 2 diabetes mellitus (T2DM). **Center**: A shared (“convergent”) mechanisms panel—oxidative stress (ROS), chronic low-grade inflammation (IL-6), mitochondrial dysfunction, altered extracellular matrix, impaired osteocyte mechanotransduction and bone remodeling imbalance—with arrows indicating that both aging and glycative stress feed into and are reinforced by these pathways. **Bottom**: Two bone silhouettes summarize the distinct structural outcomes—a porous, structurally deteriorated bone for aging (“Bone Loss & Structure Deterioration”) and a bone of preserved density but reduced quality for glycative stress (“Bone Quality Decline, Normal Mass”)—both converging on a fractured bone icon denoting the shared endpoint of increased skeletal fragility.

In bone, glycative stress results from a combination of AGEs and AGOEs, the latter requiring concomitant ROS and reactive carbonyl species (RCS) [15,18]. AGEs are irreversible products formed through the Maillard reaction, whereas AGOEs arise under conditions that incorporate ROS and reactive carbonyl species during their formation [19,20]. Glycative stress-derived deleterious effects on skeletal health have been associated with osteoporosis and bone fragility, particularly in aging and metabolic disorders such as type 2 diabetes (T2DM). Unlike aging, AGE accumulation primarily compromises bone quality rather than necessarily reducing bone mass. In glycative stress, particularly in T2DM, bone mineral density and even bone mass may remain unchanged or increased, yet fracture risk is paradoxically increased [21,22]. Although AGE accumulation has been associated with reductions in trabecular thickness, trabecular bone volume, and increases in cortical porosity, these structural changes are generally less pronounced than those observed during aging [23]. Consequently, whereas aging predominantly leads to skeletal fragility through a progressive loss of bone mass and microarchitectural deterioration, glycative stress contributes to fracture susceptibility mainly through deterioration of bone material properties, impaired mechanotransduction, and altered bone remodeling, explaining the disproportionately high fracture risk observed in elderly individuals and patients with T2DM despite preserved bone mineral density [21,22]. Aging and glycative stress share several molecular mechanisms that contribute to skeletal deterioration, including oxidative stress, chronic inflammation, mitochondrial dysfunction, and cellular senescence [18,24,25]. Importantly, both conditions may also converge on processes that are central to bone mechanoadaptation, including extracellular matrix alterations, impaired osteocyte mechanotransduction, dysregulated osteoblast–osteoclast communication, and altered bone remodeling [18,24]. Nevertheless, these similarities coexist with distinct initiating factors and molecular pathways. Glycative stress is characterized by the accumulation of AGEs and their interaction with the receptor for AGEs (RAGE)—an immunoglobulin-superfamily receptor encoded by the AGER gene [26]—which can modify bone matrix properties and cellular signaling. In contrast, aging involves additional processes such as cellular senescence, hormonal alterations, and progressive deterioration of the lacunocanalicular network that are not necessarily attributable to glycative stress. Thus, although aging and glycative stress may ultimately produce overlapping skeletal phenotypes, the mechanisms underlying these effects are not completely interchangeable (Figure 1).

Although bone aging, diabetes-associated skeletal fragility, AGE–RAGE signaling, and osteocyte mechanotransduction have each been previously reviewed, these processes have largely been considered separately. Consequently, an integrated comparison of the mechanisms shared by aging and glycative stress, as well as those that distinguish them, remains limited. This distinction is particularly relevant because glycative stress increases with aging and is markedly exacerbated by metabolic disorders such as T2DM, creating a biological context in which age-related and glycation-related insults can coexist and potentially reinforce one another. The present review addresses this gap by examining aging and glycative stress within a common bone-centered framework, with particular emphasis on their effects on the extracellular matrix, osteocyte mechanotransduction, intracellular mechanotransduction pathways, bone-cell communication, and remodeling. We specifically consider oxidative and inflammatory signaling, AGE accumulation and AGE–RAGE signaling, alterations in matrix properties, integrin and focal adhesion signaling, calcium/Piezo1 and Wnt pathways, YAP/TAZ signaling, and RANKL/OPG regulation as potential points of convergence, while distinguishing these from mechanisms that are more characteristic of aging or glycative stress. By integrating these mechanisms across bone cells, matrix properties, and mechanical responsiveness, this review aims to move beyond previous narrative reviews that have primarily examined bone aging, diabetes-associated skeletal fragility, AGE biology, or osteocyte mechanotransduction independently, providing a comparative framework to identify both shared therapeutic targets and condition-specific mechanisms underlying skeletal fragility.

The following sections will comparatively examine the mechanisms underlying skeletal deterioration associated with aging and glycative stress, highlighting both their shared and distinct effects across multiple levels of bone biology.

## 2. Effects of Aging and Glycative Stress on Osteocyte Viability

Osteocytes are particularly vulnerable to both aging and glycative stress because they are long-lived cells, in contrast to osteoclasts and osteoblasts [3], and are embedded within the mineralized bone matrix, which can progressively accumulate AGEs during aging, diabetes, and chronic hyperglycemia [26,27]. As the primary regulators of bone remodeling and mechanosensation, alterations in osteocyte viability and function have major consequences for skeletal homeostasis. Aging and glycative stress both impair osteocyte survival, promote bone resorption, and disrupt bone remodeling, although they do so through partly distinct mechanisms.

Convergent Effects of Aging and Glycative Stress

**Figure 2:**
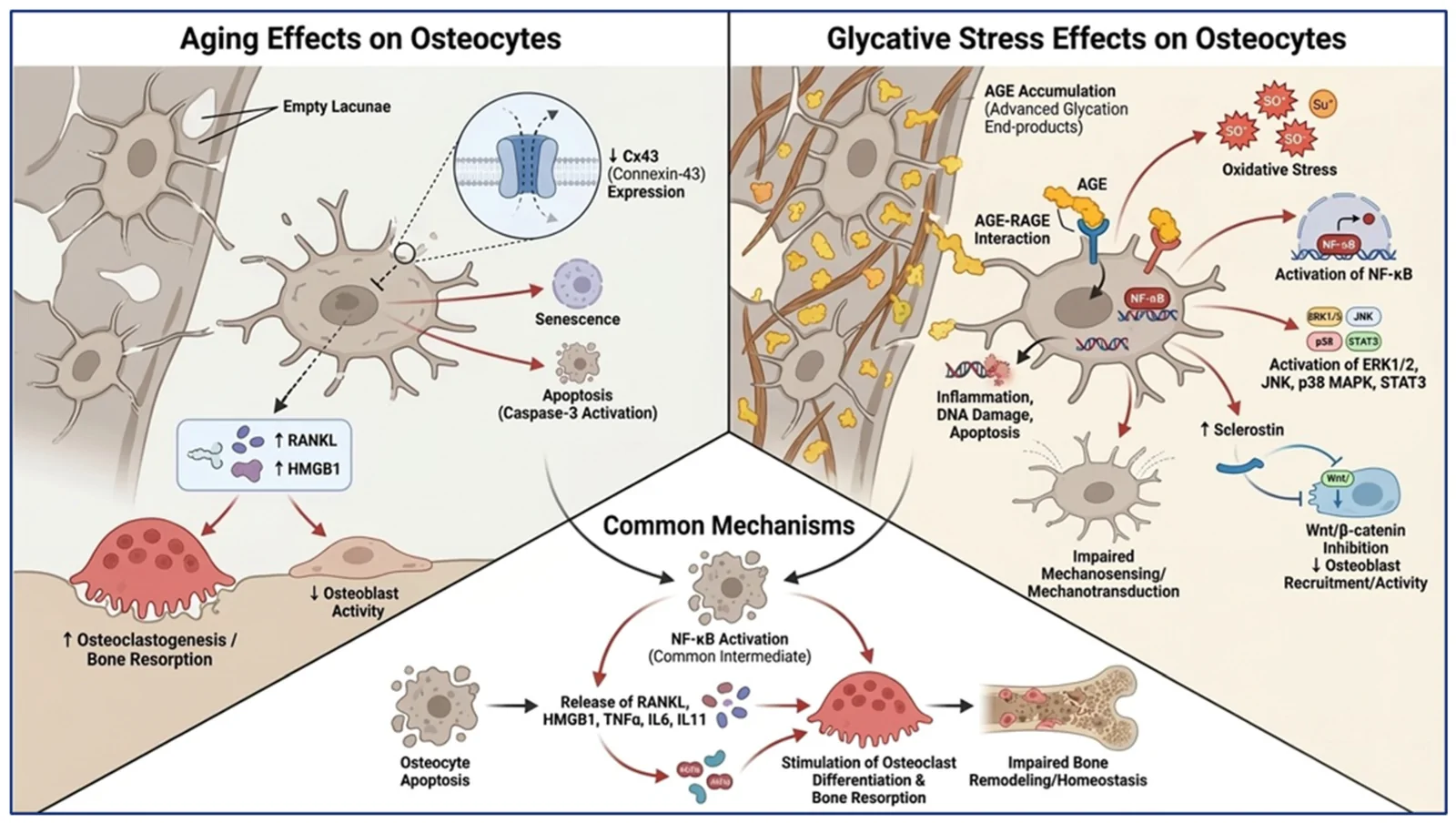
Aging and glycative stress converge on osteocyte apoptosis and NF-κB-driven osteoclast activation. Comparison of aging- and glycative stress-induced alterations in osteocyte biology and their downstream effects on bone remodeling. **Left triangle** (“Aging Effects on Osteocytes”): An osteocyte network embedded in bone matrix shows empty lacunae, denoting osteocyte loss; the inset magnification depicts reduced expression of the gap-junction protein connexin-43 (Cx43) at a dendritic contact. The affected osteocyte undergoes senescence and caspase-3-mediated apoptosis, releasing RANKL and HMGB1, which respectively increase osteoclastogenesis/bone resorption and decrease osteoblast activity. **Right triangle** (“Glycative Stress Effects on Osteocytes”): AGEs accumulate within the collagen matrix surrounding the osteocyte and engage in an AGE–RAGE interaction at the cell membrane, triggering oxidative stress, activation of NF-κB, and activation of ERK1/2, JNK, p38 MAPK and STAT3. These pathways converge on inflammation, DNA damage and apoptosis, impaired mechanotransduction, and increased sclerostin expression, which inhibits Wnt/β-catenin signaling and thereby reduces osteoblast recruitment and activity. **Bottom triangle** (“Common Mechanisms”): Apoptotic osteocytes from either pathway release RANKL, HMGB1, TNFα, IL-6 and IL-11, which converge on NF-κB activation as a shared intermediate, driving osteoclast differentiation and bone resorption and ultimately impairing bone remodeling/homeostasis.

Aging and glycative stress share several pathogenic mechanisms that ultimately compromise osteocyte viability and bone integrity. Both conditions increase osteocyte apoptosis associated with oxidative stress, mitochondrial dysfunction, and cellular senescence, and lead to deterioration of the osteocyte network and impaired bone homeostasis [26,27,28,29]. Apoptotic osteocytes stimulate osteoclastogenesis through activation of Nuclear Factor kappa B (NF-κB) signaling, triggering pro-resorptive mediators such as RANKL, HMGB1, and the inflammatory cytokines TNFα, IL-6 and IL-11 [30,31]. HMGB1 released from apoptotic osteocytes promotes osteoclast differentiation directly through RAGE signaling and indirectly by inducing neighboring osteocytes to express RANKL and pro-inflammatory factors [31]. Both aging- and glycative stress-related osteocyte dysfunction are associated with increased osteoclast activity, reduced osteoblast functions, and impaired regulation of bone remodeling (Figure 2). Furthermore, high or sustained activation of AGE–RAGE and HMGB1/RAGE signaling shifts osteocytes from adaptive responses toward apoptosis, inflammation, and enhanced bone resorption.

Divergent Effects of Aging and Glycative Stress

Aging is characterized by progressive structural deterioration of the osteocyte network. Aged osteocytes exhibit a rounded morphology, reduced dendritic processes, lacunocanalicular network degeneration, empty lacunae, reduced cellular connectivity, hormonal changes and the accumulation of cellular damage over time [24]. These age-related alterations are closely associated with apoptosis and are accompanied by increased cellular senescence and accumulation of molecular damage over time [28]. Aging also reduces the expression of Connexin-43 (Cx43), a key mediator of osteocyte communication [32,33]. Loss of Cx43 during aging promotes osteocyte apoptosis and decreases pro-survival signaling. In this regard, Cx43 silencing in osteocytic cells triggers caspase-3-mediated apoptosis along with upregulation of apoptosis-related genes and reduces the levels of the pro-survival signaling axis miR-21/Phosphatase and TENsin homolog (PTEN)/Akt. Following this, apoptotic osteocytes increase the expression of RANKL and HMGB1, thereby enhancing osteoclastogenesis and cortical bone resorption [34] (Figure 2).

Glycative stress exerts its effects primarily through AGE accumulation within the bone matrix and activation of AGE–RAGE signaling [26]. In contrast to aging, glycative stress introduces additional mechanisms related to the accumulation of AGEs within the bone matrix, AGE–RAGE signaling, chronic hyperglycemia, and reactive carbonyl and oxidative stress. The effects of RAGE ligands on bone cells are biphasic: Low or transient AGE/HMGB1 doses stimulate osteoblast autophagy and differentiation, whereas high or sustained doses induce osteoblast/osteocyte apoptosis and increase cytokine production that promotes osteoclastogenesis. AGE binding to RAGE activates ROS generation, NF-κB, ERK1/2, JNK, p38 MAPK, and STAT3 pathways, resulting in oxidative stress, inflammation, DNA damage, and apoptosis in osteocyte-like cells. The resulting cellular dysfunction impairs the ability of osteocytes to sense and respond to mechanical stimuli. Therefore, unlike aging, glycative stress directly hinders osteocyte mechanotransduction, leading to altered regulation of bone remodeling [26,27]. In addition, glycative stress directly increases sclerostin expression, a key osteocyte-derived inhibitor of canonical Wnt/β-catenin signaling, thereby suppressing mechanically dependent osteoblast recruitment and activity [35] (Figure 2). Therefore, glycative stress introduces a metabolically driven, AGE–RAGE-dependent component of osteocyte injury that is distinct from the predominantly structural and degenerative changes observed during aging.

## 3. Effects of Aging and Glycative Stress on Osteoblast and Osteoclast Dysfunction

Aging and glycative stress are major contributors to skeletal fragility because they disrupt the balance between bone formation and bone resorption. Both conditions impair osteoblast function, alter osteoblast-osteoclast communication, and compromise bone remodeling. While they share several downstream consequences, including reduced bone formation and increased bone fragility, the underlying mechanisms differ substantially.

Convergent Effects of Aging and Glycative Stress

Aging and glycative stress converge on several pathways that impair bone remodeling. In both conditions, osteoblasts exhibit increased oxidative stress, which activates intrinsic apoptotic pathways through modulation of the Bcl-2 family of proteins (increased Bax/Bcl-2 ratios), mitochondrial membrane depolarization (causing cytochrome c release), and caspase-3 activation, thus promoting osteoblast apoptosis [26,27,36].

**Figure 3:**
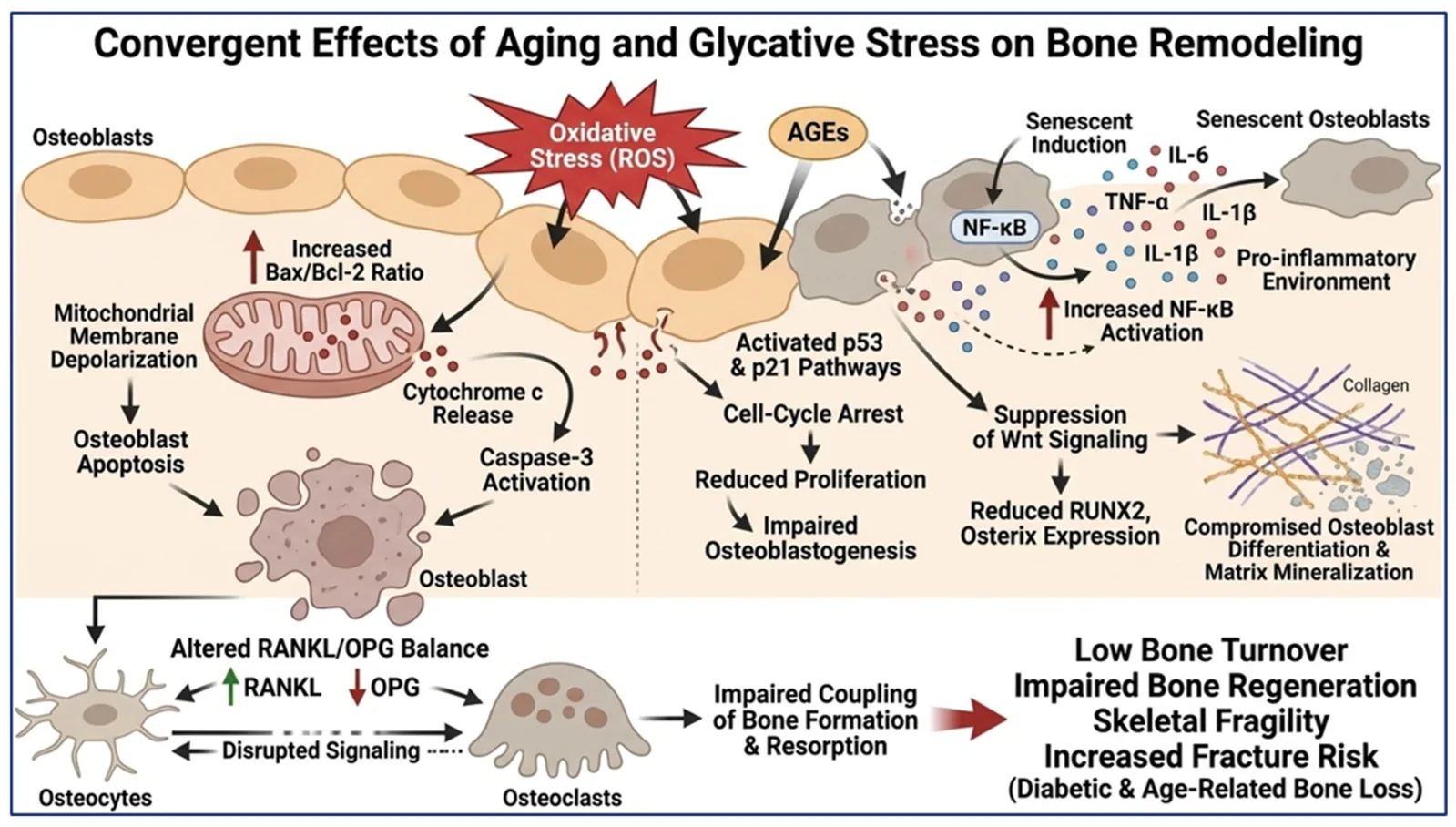
Convergent effects of aging and glycative stress on osteoblast apoptosis, senescence and matrix mineralization. Shared downstream pathways by which oxidative stress and AGE accumulation compromise osteoblast function and bone remodeling. A row of osteoblasts is exposed to oxidative stress (ROS) and AGEs, which activate three parallel branches. **Left branch**: An increased Bax/Bcl-2 ratio causes mitochondrial membrane depolarization and cytochrome c release, activating caspase-3 and driving osteoblast apoptosis. **Center branch**: Activation of p53 and p21 induces cell-cycle arrest and reduced proliferation, impairing osteoblastogenesis. **Right branch**: Senescence induction increases NF-κB activation and secretion of the pro-inflammatory cytokines IL-6, TNF-α and IL-1β, generating a pro-inflammatory environment and senescent osteoblasts; the same NF-κB/inflammatory signaling suppresses Wnt signaling, reducing RUNX2 and osterix expression and yielding a disorganized, poorly crosslinked collagen matrix (“Compromised Osteoblast Differentiation & Matrix Mineralization”). **Bottom**: Osteoblast apoptosis alters the RANKL/OPG balance (increased RANKL, decreased OPG) and disrupts osteocyte–osteoclast signaling, impairing the coupling of bone formation and resorption and culminating in low bone turnover, impaired bone regeneration, skeletal fragility and increased fracture risk characteristic of both diabetic and age-related bone loss.

Both processes also trigger a senescence-associated secretory phenotype (SASP) that leads to a pro-inflammatory environment characterized by increased NF-κB activation and secretion of cytokines such as IL-6, TNF-α, and IL-1β and other inflammatory mediators that further inhibit osteogenesis and enhance osteoclastogenic signaling [37,38]. Growing evidence also suggests that apoptotic and senescence-related pathways involve activation of p53 and its downstream target p21, resulting in cell-cycle arrest, reduced proliferation, and impaired osteoblastogenesis [28,39,40]. These changes are accompanied by suppression of major osteogenic pathways, particularly Wnt signaling, and reduced expression of key osteogenic transcription factors such as RUNX2 and Osterix, further compromising osteoblast differentiation and matrix mineralization [27,41,42,43,44] (Figure 3).

In addition, aging and glycative stress alter the RANKL/OPG balance and disrupt communication among osteoblasts, osteocytes, and osteoclasts, impairing the coupling between bone formation and resorption [41,44]. Altogether, these mechanisms are proposed to contribute to the low bone turnover, impaired bone regeneration, skeletal fragility and increased fracture risk that are hallmarks of both diabetic and age-related bone loss [45,46,47] (Figure 3).

Divergent Effects of Aging and Glycative Stress

**Figure 4:**
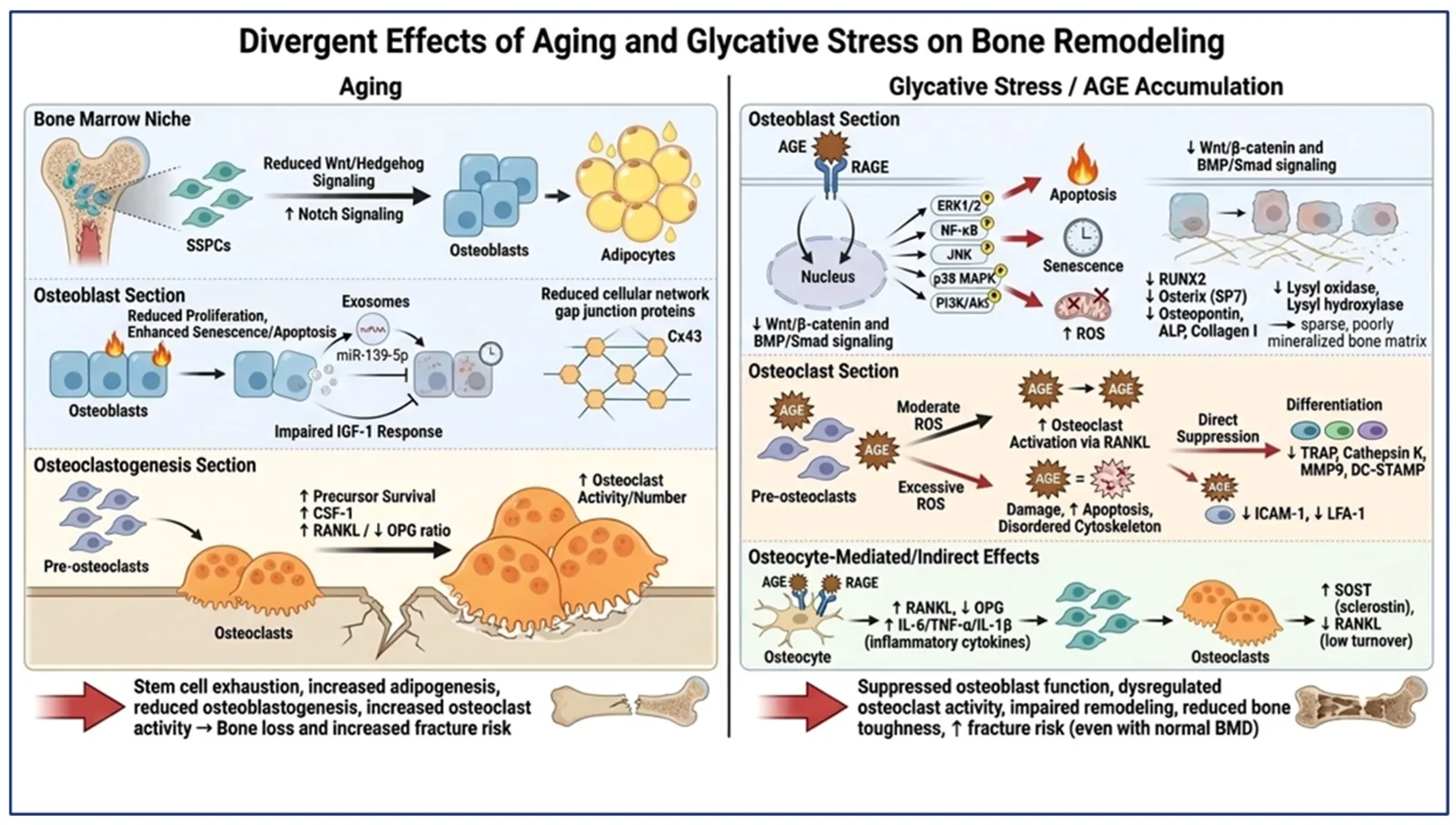
Divergent cellular and molecular mechanisms by which aging and glycative stress dysregulate osteoblasts and osteoclasts. Side-by-side comparison of aging-specific (**left**) and glycative stress/AGE-specific (**right**) effects on bone-forming and bone-resorbing cells. Aging (**left**): In the bone marrow niche, skeletal stem/progenitor cells (SSPCs) show reduced Wnt/Hedgehog and increased Notch signaling, skewing differentiation away from osteoblasts and toward adipocytes; in the osteoblast section, aged osteoblasts display reduced proliferation and enhanced senescence/apoptosis and secrete miR-139-5p-enriched exosomes that impair IGF-1 responsiveness in neighboring cells, accompanied by reduced Cx43 gap-junction connectivity; in the osteoclastogenesis section, increased precursor survival, elevated CSF-1 and a raised RANKL/OPG ratio increase osteoclast activity and number, resorbing bone. These changes are summarized as stem-cell exhaustion, increased adipogenesis, reduced osteoblastogenesis and increased osteoclast activity, leading to bone loss and increased fracture risk. Glycative stress/AGE accumulation (**right**): In the osteoblast section, AGE–RAGE engagement activates ERK1/2, NF-κB, JNK, p38 MAPK and PI3K/Akt, driving apoptosis, senescence and increased ROS, while downregulated Wnt/β-catenin and BMP/Smad signaling reduce RUNX2, osterix (SP7), osteopontin, ALP and collagen I, and reduced lysyl oxidase/lysyl hydroxylase yield a sparse, poorly mineralized matrix; in the osteoclast section, AGE-exposed pre-osteoclasts show moderate ROS enhancing RANKL-driven activation, whereas excessive ROS causes damage, disordered cytoskeleton and apoptosis, and AGEs directly suppress differentiation markers (TRAP, cathepsin K, MMP9, DC-STAMP, ICAM-1, LFA-1); in the osteocyte-mediated/indirect-effects section, AGE–RAGE signaling on osteocytes increases RANKL, IL-6/TNF-α/IL-1β and decreases OPG, stimulating osteoclasts, whereas an alternative low-turnover phenotype shows increased sclerostin (SOST) with decreased RANKL. These divergent effects are summarized as suppressed osteoblast function, dysregulated osteoclast activity, impaired remodeling, reduced bone toughness and increased fracture risk despite normal BMD.

The predominant effect of aging on osteoblasts is the progressive deterioration of skeletal stem cell progenitor (SSPC) function and the bone marrow niche. Aging reduces the pool of skeletal stem/progenitor cells and shifts their differentiation from osteoblasts toward adipocytes [48]. This process is driven by decreased Wnt and Hedgehog signaling activities and reduced osteopontin expression, collectively resulting in reduced osteoblastogenesis and increased marrow adiposity [41,42,43]. In contrast, aging has been associated with increased Notch signaling in SSPCs, promoting adipogenic rather than osteogenic differentiation. In these cells, inhibition of Notch restores osteoblast formation, reduces marrow adiposity, and improves bone mass and repair, identifying Notch modulation as a potential strategy to counteract skeletal aging [49]. Aging is also associated with reduced IGF-1 levels and IGF-1 resistance in osteoblasts, attenuating their proliferative and anti-apoptotic actions in these cells [41,42]. Interestingly, communication of osteoblasts with surrounding skeletal cells is also impaired in aging bone. This process is based on substantial alterations in the molecular cargo of extracellular vesicles secreted by osteoblasts during aging [8]. For example, it has been reported that aged osteoblasts suppress the proliferation of neighboring bone-forming cells by promoting cellular senescence and apoptosis through the release of exosomes enriched in miR-139-5p [41] (Figure 4).

Aging also promotes osteoclastogenesis through increased survival of osteoclast precursors [50], elevated levels of colony-stimulating factor 1 (CSF-1) [51], and an imbalance in the RANKL/OPG system towards bone resorption that is characterized by increased RANKL and decreased OPG expression [41]. Additionally, age-related alterations in regulatory proteins such as Connexin 43 (Cx43) signaling can promote osteoclast formation and activity [34], and defects in Sonic hedgehog signaling (Shh) may contribute to the impaired bone formation and resorption observed in the age-related delay of fracture healing [52]. Thus, age-related bone loss is driven primarily by stem-cell exhaustion, altered lineage commitment, deterioration of the bone marrow microenvironment, and increased osteoclast activity (Figure 4).

As noted in the previous sections, unlike aging, glycative stress is primarily a consequence of AGE accumulation and AGE–RAGE signaling. Activation of RAGE triggers oxidative stress, inflammation, and intracellular signaling pathways including ERK1/2, JNK, p38 MAPK, PI3K/Akt, and NF-κB. Although low concentrations of AGEs may transiently stimulate autophagy through the RAGE/Raf/MEK/ERK pathway and promote short-term cell survival due to cell self-degradation of damaged components, persistent AGE exposure induces mitochondrial damage, DNA damage, apoptosis, and senescence in osteoblasts. These actions ultimately impair osteoblast viability and function, leading to increased apoptosis [26,27,53] (Figure 4).

Glycative stress directly suppresses osteoblast differentiation and function, mediated by downregulation of essential osteogenic transcription factors, including RUNX2 and osterix (SP7), together with downstream markers such as alkaline phosphatase (ALP), osteocalcin, bone sialoprotein, and type I collagen [27,44]. Some studies have reported that AGEs downregulate ALP/RUNX2/osterix/osteopontin/osteocalcin expression and promote apoptosis via altered NF-κB and TGF-β signaling, ER stress and imbalance of Bax/Bcl-2 [26,54,55,56]. Simultaneously, glycative stress inhibits Wnt/β-catenin and BMP/Smad signaling, thereby impairing osteogenic commitment and maturation [27,44]. Osteoblast-dependent matrix production under glycative stress is compromised by reduced expression of lysyl oxidase and lysyl hydroxylase, enzymes that are responsible for collagen maturation and extracellular matrix organization [44]. Consequently, osteoblasts exhibit diminished matrix deposition and mineralization capacity, leading to reduced bone formation [26,44] (Figure 4).

The effects of glycative stress on osteoclasts are more complex and context-dependent. It seems that glycative stress affects osteoclast survival and functions through oxidative stress pathways, so that moderate levels of ROS production can act as a secondary messenger in RANKL signaling that potentiates osteoclast activation. However, excessive ROS eventually induces osteoclast mitochondrial damage, cytoskeletal disorganization, impaired actin ring formation (a structure required for osteoclast bone resorption) and apoptosis [26,27,44]. Another level of glycative stress modulation involves its direct inhibitory actions on osteoclast differentiation by suppressing RANKL-induced activation of JNK, p38 MAPK, Akt, and NFATc1 signaling pathways that are essential for osteoclastogenesis. This inhibition reduces the expression of osteoclast-specific genes such as tartrate-resistant acid phosphatase (TRAP), cathepsin K, matrix metalloproteinase-9, and DC-STAMP, resulting in impaired precursor fusion and decreased formation of mature multinucleated osteoclasts. AGEs additionally suppress adhesion-related pathways involving ICAM-1 and LFA-1, which are required for osteoclast precursor migration and cell–cell fusion [44] (Figure 4).

However, these direct inhibitory effects may differ from indirect actions of glycative stress through AGE–RAGE-mediated alterations of osteocyte and osteoblast communication with osteoclasts. Activation of RAGE signaling in OCY454-12H and MLO-Y4 osteocytic cells increases RANKL expression, decreases OPG production and enhances secretion of pro-inflammatory cytokines such as TNF-α, IL-1β, and IL-6 through NF-κB-dependent mechanisms, generating a microenvironment that promotes osteoclast recruitment and activation. Furthermore, NF-κB-dependent production of inflammatory cytokines, including TNF-α, IL-1β, and IL-6, enhances the responsiveness of osteoclast precursors to RANKL and stimulates bone resorption [26,27]. Therefore, although AGEs may directly suppress osteoclast differentiation in isolated culture systems or affect osteoclast precursor differentiation, some studies suggest that the overall in vivo environment may still favor increased osteoclastic activity because of changes in bone-cell crosstalk. However, other studies in MLO-Y4-A2 osteocyte-like cells reported opposite results, showing that AGEs decrease osteocytic RANKL expression, together with increased SOST/sclerostin expression, suggesting a low-turnover phenotype that may favor the persistence and accumulation of AGEs within the bone matrix [26,35,57]. Overall, the available evidence does not support a uniform effect of AGEs on osteocytic RANKL expression. Rather, AGE-induced regulation of RANKL appears to be context- and model-dependent, with the differentiation state of osteocyte-like cells being a particularly relevant consideration. Therefore, although increased RANKL expression and a consequent promotion of osteoclastogenesis have been reported in several models, the evidence from MLO-Y4-A2 cells indicates that AGEs may also induce a low-turnover osteocytic phenotype characterized by increased SOST and reduced RANKL expression. Further studies using well-characterized, mature osteocyte models and in vivo systems are required to determine whether one of these responses predominates under physiological or pathological conditions of glycative stress. Nonetheless, the combined suppression of osteoblast activity and dysregulation of osteoclast differentiation by AGEs is believed to result in impaired bone remodeling, reduced tissue toughness, and increased fracture risk despite relatively normal bone mineral density [26,27,54,55,58,59].

## 4. Effects of Aging and Glycative Stress on Extracellular Matrix, Bone Material Properties and Mechanotransduction

Bone adapts to mechanical loading through a complex mechanotransduction system that converts physical stimuli into biochemical signals regulating osteocytes, osteoblasts, and osteoclasts [60]. Osteocytes, interconnected through the lacuno-canalicular network (LCN), are the principal mechanosensors of bone and coordinate anabolic responses through pathways involving integrins, Piezo channels, Ca^2+^ signaling, connexin 43 (Cx43), PTH1R and Wnt/β-catenin, and Hippo/YAP/TAZ [61,62]. Although both aging and glycative stress compromise bone adaptation to mechanical loading and promote skeletal fragility, they may do so through distinct mechanisms. Aging is associated with broad alterations in osteocyte viability and mechanosensory structures, whereas glycative stress predominantly affects cellular signaling and matrix properties through the accumulation of AGEs and activation of RAGE-dependent pathways.

Convergent Effects of Aging and Glycative Stress

Aging and glycative stress converge on a common phenotype of impaired skeletal mechanotransduction. In this regard, osteocyte responsiveness to mechanical loading declines due to disruption of some mechanisms that are similarly affected by these two deleterious conditions.

**Figure 5:**
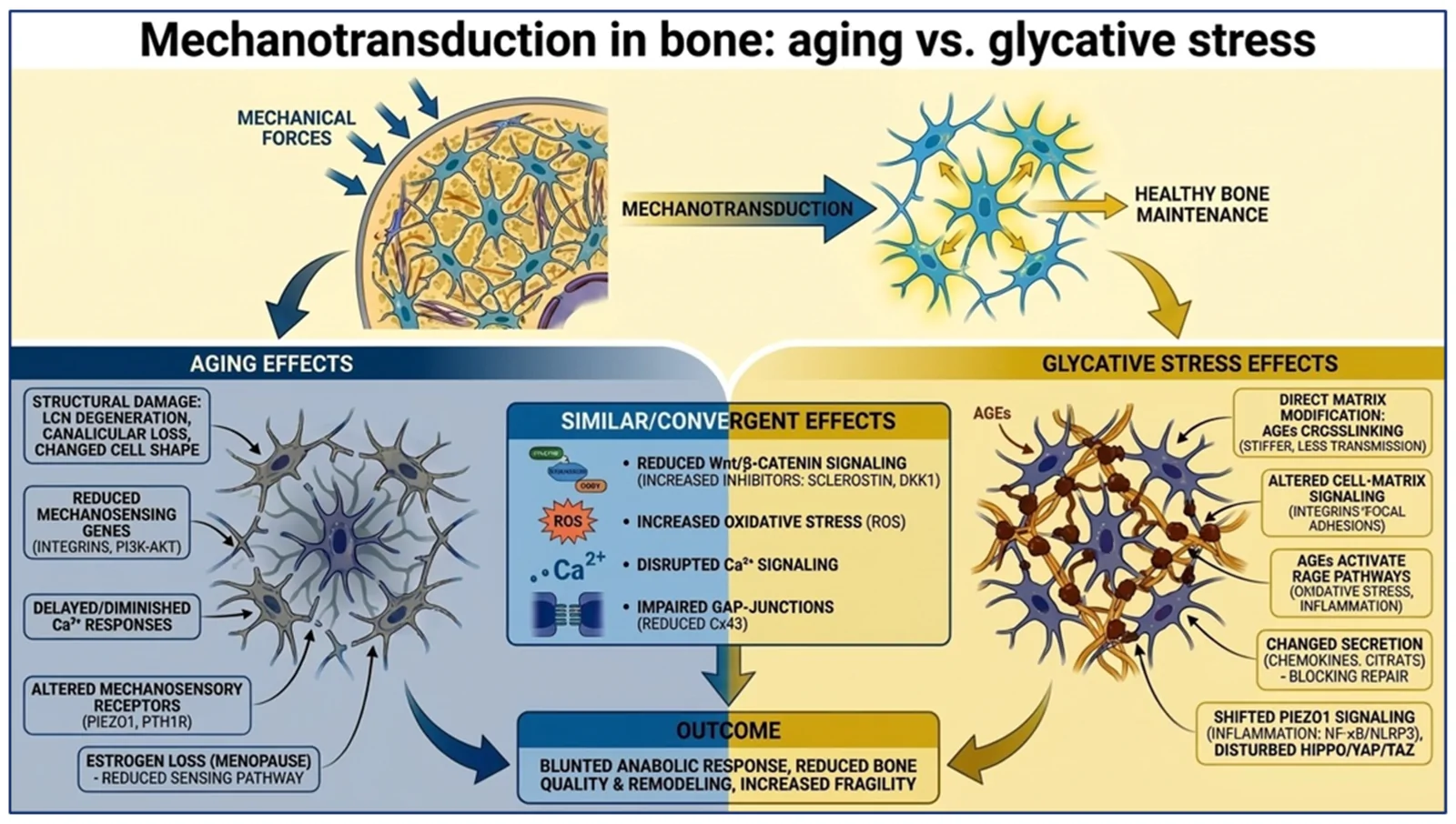
Aging versus glycative stress effects on osteocyte mechanotransduction. Comparative schematic of bone mechanotransduction under aging and glycative stress. **Top**: Mechanical forces act on a cross-section of bone containing an interconnected osteocyte lacunocanalicular network; effective mechanotransduction normally sustains a metabolically active osteocyte network supporting healthy bone maintenance. **Left** (“Aging Effects”): Structural damage from LCN degeneration, canalicular loss and altered osteocyte morphology, reduced expression of mechanotransduction genes (integrins, PI3K-Akt), delayed/diminished Ca^2+^ responses, altered mechanosensory receptors (Piezo1, PTH1R), and estrogen loss at menopause collectively reduce the sensing pathway, depicted as an osteocyte with fewer, shorter dendrites. **Right** (“Glycative Stress Effects”): Direct AGE crosslinking stiffens the matrix and reduces force transmission, altered cell–matrix/integrin–focal-adhesion signaling, RAGE pathway activation (oxidative stress, inflammation), changed secretion of chemokines and citrate that blocks repair, and shifted Piezo1 signaling toward NF-κB/NLRP3 inflammatory pathways and disturbed Hippo/YAP/TAZ pathways are depicted around an osteocyte enmeshed in crosslinked (AGE-modified) fibers. **Center**: A shared “Similar/Convergent Effects” box lists reduced Wnt/β-catenin signaling with increased inhibitors sclerostin and Dkk1, increased oxidative stress (ROS), disrupted Ca^2+^ signaling, and impaired gap junctions (reduced Cx43), feeding into a common outcome of blunted anabolic response, reduced bone quality and remodeling, and increased fragility.

The Wnt/β-catenin pathway is considered the master anabolic promoter of mechanical signals in bone [63]. Aging is associated with the downregulation of genes involved in the activation of the Wnt signaling pathway and with the increased expression of Wnt antagonists (sclerostin and Dkk1), leading to a diminished osteogenic response to mechanical stimulation in aged bone [63,64]. This deleterious scenario observed with aging seems to be exacerbated by AGE accumulation, since AGEs and high glucose synergistically increase sclerostin in MLO-Y4 osteocyte-like cells [65]. In addition, aging and glycative stress-induced oxidative stress contribute to impaired mechanotransduction by disrupting osteocyte communication and mechanosensitive signaling pathways. However, whereas in aging ROS is primarily associated with osteocyte dysfunction and loss of mechanotransduction capacity [66], in glycative stress ROS arises largely downstream of AGE–RAGE signaling and acts together with matrix modifications to impair mechanotransduction [67,68] (Figure 5).

Both aging and glycative stress impair the early osteocyte mechanotransduction response by reducing the amplitude, coordination, and efficiency of load-induced Ca^2+^ signaling, leading to diminished mechanosensitivity [69]. Thus, both conditions converge on defective Ca^2+^ signaling, but glycative stress appears to promote an inflammatory response (see below) [70,71].

Glycative stress also reduces Cx43 expression and disrupts osteocyte gap-junction communication in a manner like aging, compromising the integrity of the osteocyte network and the propagation of mechanical signals [66].

Consequently, aging and glycative stress both lead to a blunted mechano-anabolic response characterized by reduced bone formation, impaired remodeling, and increased skeletal fragility (Figure 5).

Divergent Effects of Aging and Glycative Stress

The decline in mechanosensitivity during aging is primarily driven by structural and cellular deterioration of the osteocyte network. Aging induces degeneration of the LCN, loss of canalicular density and osteocyte rounding, changes that cause reduced fluid-flow-induced shear stress, compromising mechanotransduction [72]. These structural changes are accompanied by reduced expression of mechanotransduction-related genes, including components of integrin signaling, calcium signaling, nitric oxide and prostaglandin production, and downstream pathways such as PI3K-Akt. These alterations in key mechanosensory molecules provide a plausible explanation for the decline in the mechanical responsiveness observed with aging [64,73] (Figure 5).

Furthermore, aging is associated with fewer and delayed Ca^2+^ responses during mechanical loading [69] and may involve decreased function of Piezo1, the calcium-permeable cation channel that transduces membrane tension and mechanical deformation [74], although direct evidence in osteocytes remains limited. In addition, multiple osteoblastic and osteocytic mechanosensors shown below are affected in aging: Resistance to age-related bone loss has been observed in integrin α2-deficient mice, suggesting that integrin α2β1 absence alters bone quality and age-related skeletal changes in a complex manner [75]. A mechanosensitive G-protein-coupled receptor (GPCR) in osteocytes, the PTH1R [76], relies on interactions with other mechanosensory structures, including primary cilia and caveolae, to transduce osteogenic signals [77,78]. Aging-associated alterations in ciliary structure and signaling [79], together with increased caveolin-1 expression and caveolae-mediated modulation of PTH1R activity [78], may impair receptor mechanotransduction, resulting in reduced osteocyte mechanosensitivity, diminished osteogenic gene expression, and compromised bone remodeling. Furthermore, one of the features of aging in women is the progressive decline in estrogen levels, with the most dramatic change occurring during the menopausal transition. Interestingly, mechanical stimulus can activate ERα in a ligand-independent manner, supporting bone homeostasis [80]. Given that ERα participates in the transduction of mechanical forces into pro-survival signals in osteocytes and osteoblasts [80], estrogen depletion during aging could substantially contribute to the diminished bone mechanoresponsiveness observed in postmenopausal women [81]. Collectively, these changes reduce the ability of osteocytes to detect, propagate, and respond to mechanical stimuli, resulting in diminished skeletal adaptation to loading (Figure 5).

Compared to aging, AGEs modify collagen and increase non-enzymatic crosslinking, altering matrix stiffness, toughness, viscoelasticity, and the transmission of mechanical forces through the bone matrix. These modifications disrupt integrin-mediated cell–matrix interactions and focal adhesion signaling, reducing force transmission even when the expression of mechanosensor proteins remains intact [15,18,67,82] (Figure 5).

At the cellular level, AGE–RAGE signaling exacerbates the deleterious scenario observed with aging through AGE accumulation. AGEs and high glucose synergistically modify the secretion of mechanically stimulated osteocytic chemokines (VEGF, RANTES, MIP-1α/β, MCP-1, GM-CSF), suppressing their chemoattractant activity on osteoclast precursors [83]. Experimental in vitro analyses in osteocytes have also shown that mechanically dependent secretion of citrate, a metabolite with an essential role in the organization of the bone apatite–collagen structure and in maintaining matrix quality, is abolished by high glucose pre-conditioning [84].

Regarding Ca^2+^ signaling, glycative stress attenuates its mechanically stimulated transients similarly to what occurs with aging [70]. A key divergence is that glycative stress may additionally shift Piezo1 signaling toward NF-κB/NLRP3-mediated inflammatory pathways [70,71], thus probably promoting a low-turnover, pro-inflammatory bone phenotype, whereas aging is primarily linked to impaired mechanotransduction (Figure 5).

In addition, glycative stress may dysregulate Hippo/YAP/TAZ signaling, key mechanosensitive transcriptional regulators that translate mechanical cues into gene expression programs controlling osteocyte function, osteoblast differentiation, and bone formation [85]. Glycative signaling actions on Hippo/YAP/TAZ are related to the combination of matrix stiffening and ROS-induced pathway inhibition, and it has been suggested that glycative stress can potentially uncouple Hippo/YAP/TAZ-dependent cellular transcriptional programs from mechanical demands [68]. In contrast, current evidence supporting a potential role for YAP/TAZ in the age-related decline of skeletal mechanosensitivity remains limited [85].

From a therapeutic perspective, the multiple processes that converge and diverge shown above reinforce the need for combination strategies that act simultaneously against aging and glycative stress actions on the matrix and on the cellular mechanosensing machinery.

## 5. Effects of Aging and Glycative Stress on Oxidative Stress, Metabolism, Inflammation and Senescence

Aging and glycative stress profoundly affect bone homeostasis by altering cellular metabolism, inflammatory signaling, and stress-response pathways. Osteocytes, osteoblasts, and skeletal stem cells progressively lose their capacity to maintain bone integrity as oxidative stress, mitochondrial dysfunction, and metabolic abnormalities accumulate. Although aging and glycative stress arise from different upstream processes, both promote chronic inflammation, cellular senescence, and impaired bone remodeling, ultimately contributing to skeletal fragility.

Convergent Effects of Aging and Glycative Stress

**Figure 6:**
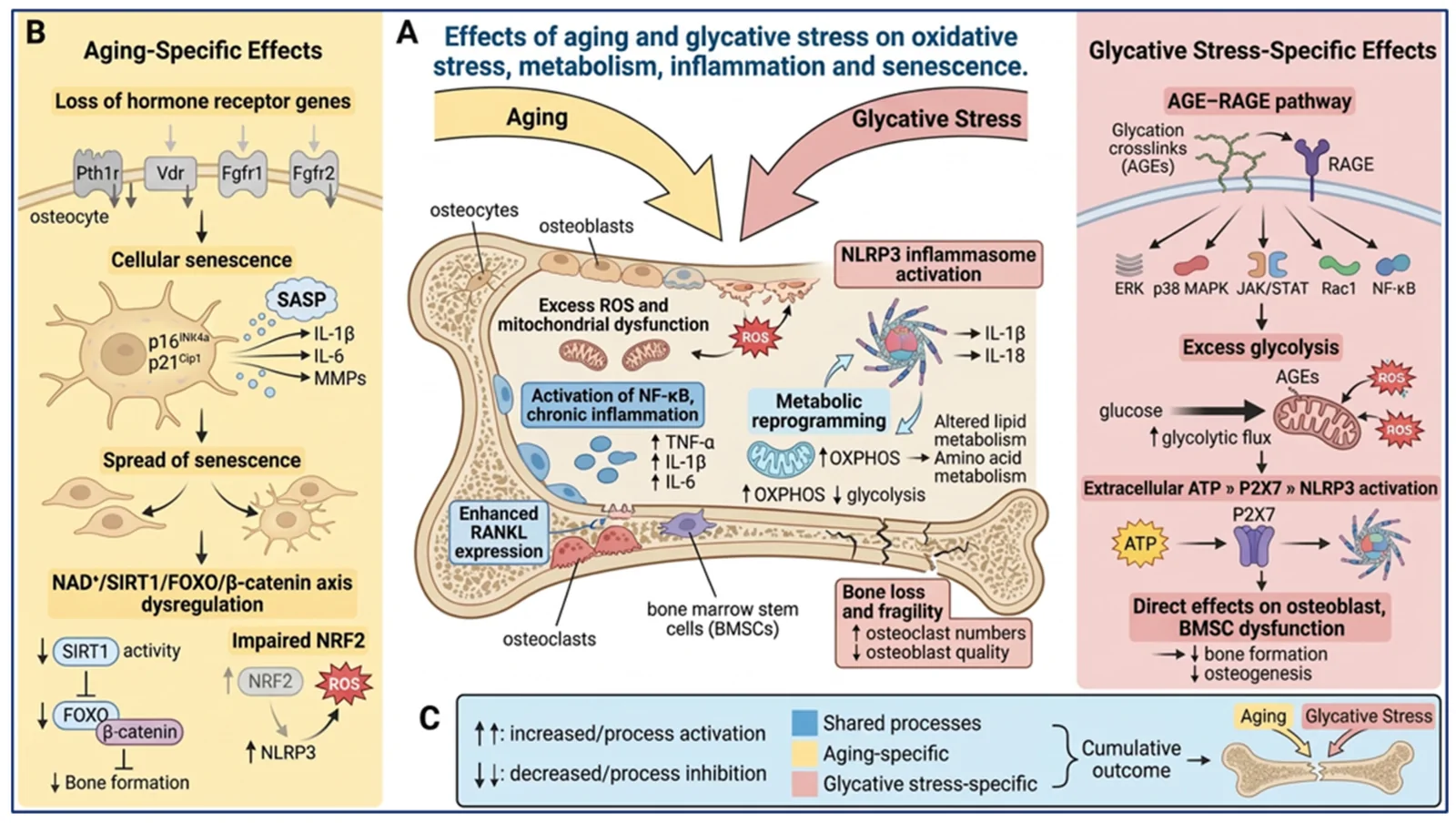
Aging- and glycative stress-specific pathways converging on inflammasome activation, metabolic reprogramming and bone loss. Three-panel schematic of oxidative, metabolic and inflammatory changes driving bone loss. Panel (**A**) (center): Aging and glycative stress both act on a bone cross-section containing osteocytes, osteoblasts, osteoclasts and bone marrow stem cells (BMSCs), producing excess ROS and mitochondrial dysfunction that activates the NLRP3 inflammasome (releasing IL-1β and IL-18) and NF-κB-driven chronic inflammation (increased TNF-α, IL-1β, IL-6); metabolic reprogramming (increased OXPHOS, decreased glycolysis, altered lipid and amino-acid metabolism) and enhanced RANKL expression at the osteoclast surface together culminate in bone loss and fragility, marked by increased osteoclast numbers and decreased osteoblast quality. Panel (**B**) (left, “Aging-Specific Effects”): Loss of hormone receptor genes (Pth1r, Vdr, Fgfr1, Fgfr2) on the osteocyte membrane triggers p16^INN4a^/p21^cip1^-driven cellular senescence with a senescence-associated secretory phenotype (SASP: IL-1β, IL-6, MMPs) that spreads to neighboring cells, alongside dysregulation of the NAD^+^/SIRT1/FOXO/β-catenin axis (decreased SIRT1, FOXO and β-catenin activity reducing bone formation) and impaired NRF2 signaling that permits ROS accumulation and NLRP3 upregulation. Panel (**C**) (right, “Glycative Stress-Specific Effects”): AGE glycation crosslinks engage RAGE, activating ERK, p38 MAPK, JAK/STAT, Rac1 and NF-κB; excess glycolytic flux increases glucose-derived AGE formation in a ROS-amplified feed-forward loop with mitochondria, and extracellular ATP acting through the P2X7 receptor further activates NLRP3, producing direct suppression of osteoblast/BMSC bone formation and osteogenesis. Legend (bottom): Arrow symbols denote increased/activated versus decreased/inhibited processes; color coding distinguishes shared processes (blue), aging-specific processes (yellow) and glycative stress-specific processes (pink), which cumulatively compromise the bone icon shown at right.

Aging and glycative stress are associated with excessive ROS production, mitochondrial dysfunction, activation of NF-κB signaling, and chronic low-grade inflammation, leading to impaired osteoblast function and enhanced osteoclastogenesis [12,26,54,55,58,86,87,88]. In both settings, oxidative stress stimulates inflammatory cytokines such as TNF-α, IL-1β, and IL-6, creating a self-amplifying inflammatory environment that disrupts bone homeostasis. These events stimulate osteoclastogenesis through enhanced RANKL expression and contribute to excessive bone resorption [89]. The AGE–RAGE pathway described previously for glycative stress and the inflammaging process observed during aging ultimately activate overlapping inflammatory mediators, suggesting that oxidative stress-driven inflammation, even though exacerbated by glycative stress, is a major point of convergence between both processes. In this regard, both aging and glycative stress converge on activation of the NLRP3 inflammasome, although the mechanisms that drive this activation seem to be different, as mentioned below. The NLRP3 inflammasome is a multiprotein innate immune complex composed of NLRP3, ASC, and caspase-1 that senses cellular stress signals such as ROS, mitochondrial dysfunction, and extracellular ATP. Its activation promotes caspase-1-mediated maturation of IL-1β and IL-18, triggering inflammatory responses that contribute to tissue degeneration and bone loss [90,91,92] (Figure 6).

Aging and glycative stress are also associated with metabolic reprogramming in bone cells. In age-related osteoporosis, bone marrow stromal stem cells (BMSCs) exhibit altered energy metabolism, including increased reliance on oxidative phosphorylation (OXPHOS), which impairs proliferation, stemness, and osteogenic differentiation. Dysregulation of lipid and amino acid metabolism, including alterations in fatty acids and tryptophan-derived kynurenine, further contributes to reduced osteoblast function in skeletal aging. In parallel, osteoclastogenesis is also metabolically regulated, with OXPHOS supporting osteoclast differentiation and glycolysis promoting mature osteoclast activation and bone resorption [93]. The metabolic rewiring observed in osteoporosis shares several features with diabetic bone disease, suggesting common mechanisms linking metabolic dysfunction to impaired bone homeostasis in both aging and glycative stress. In type 2 diabetes mellitus (T2DM), chronic hyperglycemia promotes excessive glycolytic flux, mitochondrial dysfunction, ROS accumulation, and AGE formation, which negatively affect osteoblast differentiation and survival. Compared to age-related osteoporosis, diabetic conditions induce alterations in lipid metabolism, amino acid metabolism, and mitochondrial activity, leading to impaired BMSC function and reduced osteogenesis [93,94] (Figure 6).

Overall, aging and glycative stress converge on a common network of oxidative, inflammatory, and metabolic mechanisms that contribute to skeletal deterioration.

Divergent Effects of Aging and Glycative Stress

Aging is uniquely characterized by progressive intrinsic cellular deterioration and loss of physiological resilience. Age-related changes include reduced expression of several hormone receptor genes that regulate paramount bone homeostasis functions in aged cortical bone, including parathyroid hormone receptor type 1 (Pth1r), vitamin D receptor (Vdr) and fibroblast growth factor 1 (Fgfr1) and 2 (Fgfr2), suggesting impaired hormonal responsiveness of osteocytes essential for bone homeostasis [95] (Figure 6).

Another hallmark feature of aging osteocytes includes becoming resistant to apoptosis and undergoing a distinct cell fate known as cellular senescence, driven by chronic cellular damage, telomere erosion, DNA damage, and lysosomal stress—processes related to aging rather than primarily to glycation [24,96]. Senescence in bone is a state characterized by irreversible proliferation arrest induced particularly by the cyclin-dependent kinase inhibitors p16^Ink4a^ and p21^Cip1^, changes at chromatin level and reprogramming of protein synthesis [29]. Despite the growth arrest, bone senescent cells are highly metabolically active and acquire a senescence-associated secretory phenotype (SASP), which involves the secretion of inflammatory cytokines and chemokines, such as IL-1β and IL-6, that recruit and retain immune cells, and matrix tissue-degrading proteases including metalloproteinases (MMPs) [97]. These molecules can act through paracrine and endocrine signaling pathways to promote local and systemic inflammation, immune activation, fibrosis, apoptosis, tissue damage, and stem cell dysfunction, while also facilitating the spread of senescence to neighboring and distant cells [29]. In this regard, a recent study demonstrated that osteocyte-derived RANKL is a critical mediator of age-related cortical bone loss and that its expression is enhanced by the accumulation of senescent cells within cortical bone [31]. Furthermore, recent studies have demonstrated that selective induction of caspase-8-mediated apoptosis in senescent cells, as well as suppression of the pro-inflammatory SASP with a Janus Kinase (JAK) inhibitor, attenuated bone loss, increased bone mass and strength, and improved bone microarchitecture compared with vehicle-treated mice. The skeletal benefits observed following senescent cell targeting were attributable to decreased bone resorption, accompanied by maintained bone formation in trabecular bone and increased bone formation in cortical bone [29,98]. These studies point to the key role of senescence as a deleterious process in aging bone (Figure 6).

Dysregulation of the NAD^+^/SIRT1/FOXO/β-catenin axis is a further feature of aging in bone. The NAD^+^/SIRT1/FOXO/β-catenin axis is a key metabolic pathway that preserves bone homeostasis by coordinating cellular energy status, stress responses, and osteoblast differentiation. Under physiological conditions, NAD^+^-dependent SIRT1 promotes osteogenesis by deacetylating FOXO and β-catenin, thereby enhancing Wnt/β-catenin signaling and maintaining osteocyte viability. During aging, NAD^+^ depletion, chronic inflammation, and increased mitochondrial ROS reduce SIRT1 activity, leading to FOXO activation and sequestration of β-catenin away from Wnt signaling, ultimately suppressing bone formation [99,100]. In addition, impaired SIRT1 function enhances inflammation, cellular senescence, and osteoclast activity, contributing to age-related bone loss. Restoration of NAD^+^ levels has therefore emerged as a promising strategy to preserve sirtuin activity, improve bone homeostasis, and counteract skeletal aging [99,100,101,102,103,104] (Figure 6).

As mentioned before, both aging and glycative stress converge on activation of the stress sensor system NLRP3 inflammasome. However, during aging, NLRP3 activation is associated with impaired NRF2 function. NRF2 is a redox-sensitive transcription factor that serves as a master regulator of cellular antioxidant defenses, and its dysfunction contributes to the accumulation of ROS [91]. In contrast, glycative stress causes bone matrix glycation and promotes extracellular ATP accumulation, thus favoring its binding to the purinergic receptor P2X7. The ATP–P2X7 axis then triggers NLRP3 activation [91,105]. In both NRF2 and ATP–P2x7 cases, NLRP3 activation promotes IL-1β production, establishing a self-amplifying inflammatory loop and chronic inflammatory damage [91] (Figure 6).

Compared with aging, glycative stress is primarily driven by AGE–RAGE interactions that directly activate multiple signaling pathways, including ERK, p38 MAPK, JAK/STAT, Rac1, and NF-κB, generating a self-amplifying cycle of oxidative stress and inflammation [54,55,58,68,86,87]. Glycative stress introduces an additional metabolic burden through excessive glycolytic flux, AGE formation, mitochondrial dysfunction, and post-translational modification of regulatory proteins. These processes impair osteoblast differentiation and survival, alter stem-cell function, and promote inflammatory signaling independently of chronological aging [89]. AGE–RAGE signaling also enhances cytokine production, oxidative damage, and osteoclastogenic stimuli, amplifying skeletal dysfunction. Unlike aging, which primarily reflects progressive loss of cellular resilience, glycative stress represents a metabolically driven insult that accelerates many aging-like mechanisms. Therefore, chronic hyperglycemia and AGE accumulation provide a sustained source of oxidative and inflammatory stress that can intensify inflammasome activation, senescence and disruption of bone-cell function [89].

## 6. Effects of Aging and Glycative Stress on Autophagy, Ferroptosis and Mitochondrial Dysfunction

Maintenance of bone integrity depends on efficient cellular quality-control mechanisms, particularly autophagy, which removes damaged proteins and organelles and preserves mitochondrial function. Both aging and glycative stress impair these protective pathways, leading to oxidative stress, mitochondrial dysfunction, and progressive loss of osteoblast and osteocyte viability. In addition, growing evidence suggests that ferroptosis, an iron-dependent form of lipid peroxidation-driven cell death, may contribute to bone fragility in both aged and diabetic bone.

Convergent Effects of Aging and Glycative Stress

**Figure 7:**
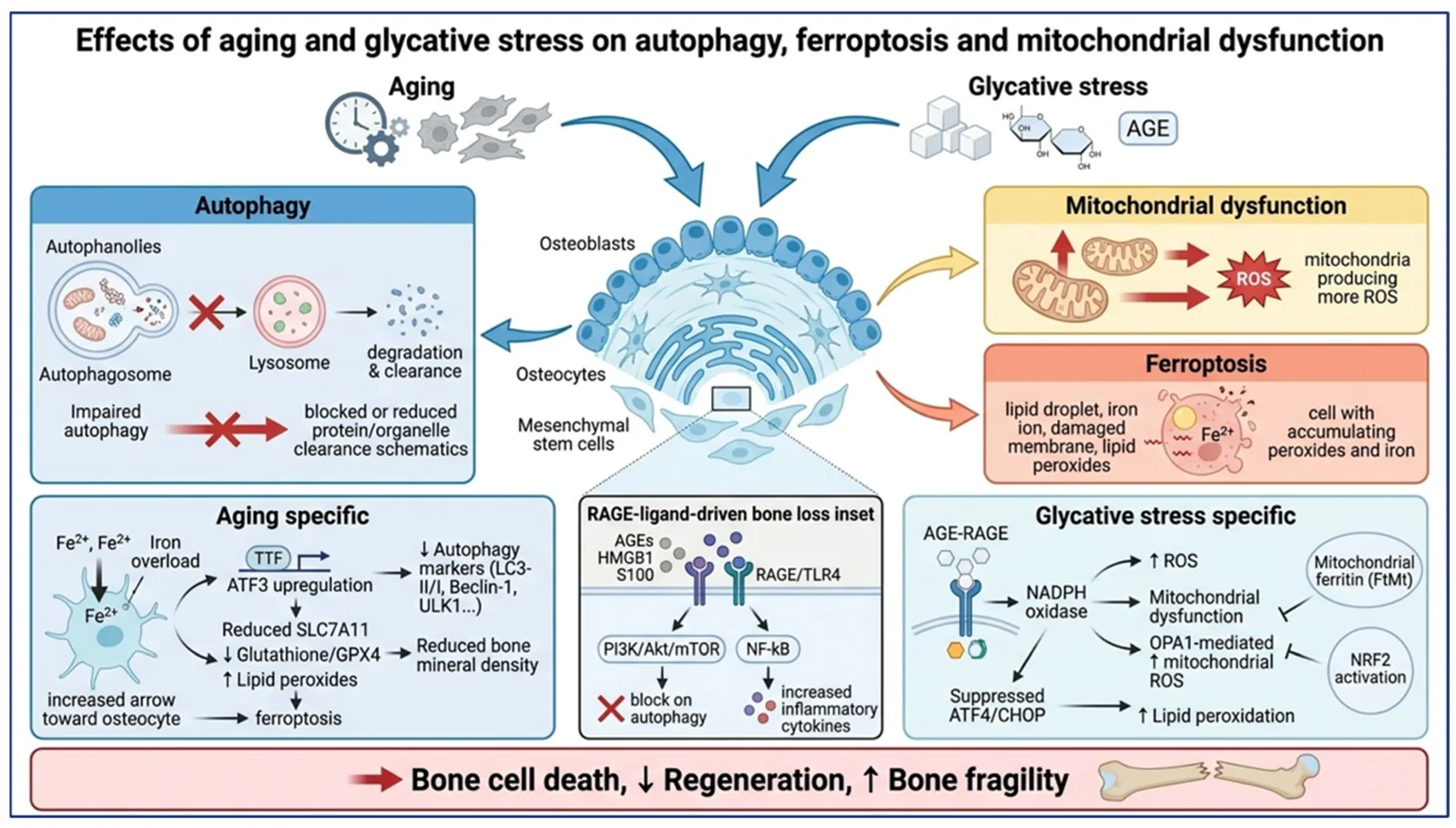
Aging and glycative stress impair autophagy and promote ferroptosis through mitochondrial dysfunction. Integrated view of autophagic, mitochondrial and ferroptotic pathways linking aging and glycative stress to bone cell death. Aging and glycative stress both converge on a bone-lining unit comprising osteoblasts, osteocytes and mesenchymal stem cells, from which four processes radiate. Autophagy (**top left**): A damaged organelle is engulfed by an autophagosome that fuses with a lysosome for degradation and clearance; impaired autophagy instead leads to reduced protein/organelle clearance. Mitochondrial dysfunction (**top right**, in yellow colour): Damaged mitochondria generate excess ROS. Ferroptosis (**top right**, in red colour): A cell accumulates a lipid droplet, free iron (Fe^2+^), a damaged membrane and lipid peroxides. Aging-specific pathway (**bottom left**): Iron overload upregulates ATF3, reducing the cystine transporter SLC7A11 and glutathione/GPX4 activity, increasing lipid peroxides and ferroptosis while lowering autophagy markers (LC3-II/I, Beclin-1, ULK1), which is linked to reduced bone mineral density. Glycative stress-specific pathway (**bottom right**): AGE–RAGE signaling activates NADPH oxidase, increasing ROS and mitochondrial dysfunction; OPA1-mediated mitochondrial ROS suppresses protective ATF4/CHOP signaling and increases lipid peroxidation, counteracted by mitochondrial ferritin (FtMt) and NRF2 activation. Center inset (“RAGE-ligand-driven bone loss”): AGEs, HMGB1 and S100 proteins engage RAGE/TLR4 receptors, activating PI3K/Akt/mTOR (blocking autophagy) and NF-κB (increasing inflammatory cytokine production). Bottom banner summarizes the convergent outcome: increased bone cell death, decreased regeneration and increased bone fragility.

Autophagy plays a dual role in the response of bone cells to glycative stress. First, it provides the principal pathway for the clearance of AGE-modified proteins and damaged organelles, such as dysfunctional mitochondria, which are removed through selective mitophagy. Second, at the same time, autophagy itself is modulated by AGEs in a concentration- and time-dependent manner. Low or transient exposure tends to elicit a cytoprotective autophagic response, whereas sustained or high-dose exposure overwhelms the system and shifts the balance towards apoptotic cell death [68,87,106,107]. AGEs activate RAGE directly and promote the release of DAMPs (damage-associated molecular patterns) such as HMGB1 and S100 proteins that activate Toll-like receptor 4 (TLR4). TLR4 signaling suppresses osteoblast autophagy through activation of the PI3K/Akt/mTOR pathway and enhances NF-κB-dependent inflammatory cytokine production. Therefore, TLR4 acts as an important downstream amplifier of AGE-induced inflammation and autophagy impairment in bone [108] (Figure 7).

In osteoblasts, under moderate oxidative stress, activation of the MAPK/FOXO3, SIRT1/FOXO3, and AMPK pathways, together with inhibition of the Akt/mTOR pathway, stimulates autophagy, facilitating the removal of excess ROS and thereby promoting osteoblast survival and bone formation [56,109]. With aging, osteocytes exhibit elevated apoptosis and reduced autophagic activity, as indicated by decreased levels of different autophagy markers (the LC3-II/I ratio, Beclin-1, ULK1...). The reduction in autophagy markers has been related to a reduction in bone mineral density in the proximal tibia, suggesting that impaired osteocyte autophagy may contribute to age-related bone loss, and that reduced autophagic activity may play a role in the development of senile osteoporosis [110] (Figure 7).

Based on the previously mentioned observations, a “RAGE-ligand-driven bone loss” paradigm has been proposed [26]. According to this paradigm, the diverse ligands that accumulate in the glycative, oxidative and inflammatory environment of aged or diabetic bone—AGEs, HMGB1 and S100 proteins—converge on a shared intracellular network that reduces osteoblast and osteocyte viability while amplifying osteoclastogenic cytokine output, regardless of the receptor through which signaling is initiated [26] (Figure 7).

A further point of convergence is ferroptosis. This process is an iron-dependent form of regulated cell death characterized by the lethal accumulation of lipid peroxides and the failure of glutathione peroxidase 4 (GPX4)-mediated repair. Unlike classical apoptosis, ferroptosis does not require caspase activation; it depends instead on the balance between iron availability, polyunsaturated fatty acid (PUFA)-containing membrane lipids and the GPX4/glutathione antioxidant system [111]. In bone, increasing evidence suggests that ferroptosis represents a common downstream pathway linking aging, glycative stress, and diabetes-associated skeletal deterioration. Both conditions promote oxidative stress, mitochondrial dysfunction, iron dysregulation, and impaired antioxidant defenses, ultimately leading to osteoblast, osteocyte, and mesenchymal stem cell dysfunction [111,112]. The interaction between ferroptosis and metabolic reprogramming may be particularly important. Diabetes-induced ferroptosis and aging-associated reductions in cellular metabolic fitness synergistically impair BMSC osteoblastogenesis and bone regeneration. Consequently, ferroptosis emerges as a central mechanism connecting cellular aging, metabolic stress, and reduced skeletal regenerative capacity [111,112]. Although ferroptosis represents a plausible point of convergence between aging and glycative stress, the underlying mechanisms involved in these two deleterious processes differ to some extent (Figure 7).

Divergent Effects of Aging and Glycative Stress

Ferroptosis contributes to age-related cortical bone loss by promoting osteocyte death through iron overload and lipid peroxidation by inducing activating transcription factor 3 (ATF3). ATF3 simultaneously increases cellular iron uptake via transferrin receptor 1 (TfR1) and suppresses the cystine transporter SLC7A11 [113]. Reduced cystine availability limits glutathione synthesis, decreases GPX4 activity, and enhances lipid peroxide accumulation, ultimately triggering ferroptosis [113]. Age-related iron overload therefore acts together with impaired antioxidant defenses to promote osteocyte loss and skeletal fragility [113] (Figure 7).

Chronic glycative stress promotes ferroptosis primarily through AGE accumulation and sustained RAGE signaling. AGE–RAGE activation stimulates NADPH oxidase-derived ROS production and mitochondrial oxidative stress, creating a cellular environment favorable for ferroptotic death [111]. Mitochondrial dysfunction, particularly through OPA1-mediated increases in mitochondrial ROS and suppression of protective ATF4/CHOP signaling, may further sensitize glycated osteoblasts and osteocytes to ferroptosis [112]. Moreover, hyperglycemia enhances iron-dependent lipid peroxidation and ferroptotic cell death, impairing osteoblast viability and osteogenic differentiation. Protective mechanisms include mitochondrial ferritin (FtMt), which restores iron homeostasis, and NRF2 activation, which suppresses ferroptosis and preserves osteoblast function [114,115] (Figure 7).

Collectively, these findings identify impaired autophagy, mitochondrial dysfunction, and ferroptosis as interconnected mechanisms through which aging and glycative stress converge to reduce bone cell survival, impair skeletal regeneration, and promote bone fragility, despite involving partially distinct upstream molecular pathways.

## 7. Therapies for Age/Glycative Stress-Related Bone Loss

The therapeutic implications of skeletal aging and glycative stress should be considered according to the level of evidence supporting each intervention. Current clinical therapies primarily target established mechanisms of osteoporosis, whereas strategies specifically directed against glycative stress, cellular senescence, AGE–RAGE signaling, autophagy, or ferroptosis remain largely experimental. Accordingly, the following approaches are discussed separately according to their degree of clinical and preclinical validation.

### 7.1. Established Clinical Treatments

Several pharmacological treatments are currently established for the prevention and treatment of osteoporosis and are particularly relevant to age-related skeletal deterioration. These include anabolic agents targeting PTH1R, anti-sclerostin therapy, and anti-resorptive drugs.

**Figure 8:**
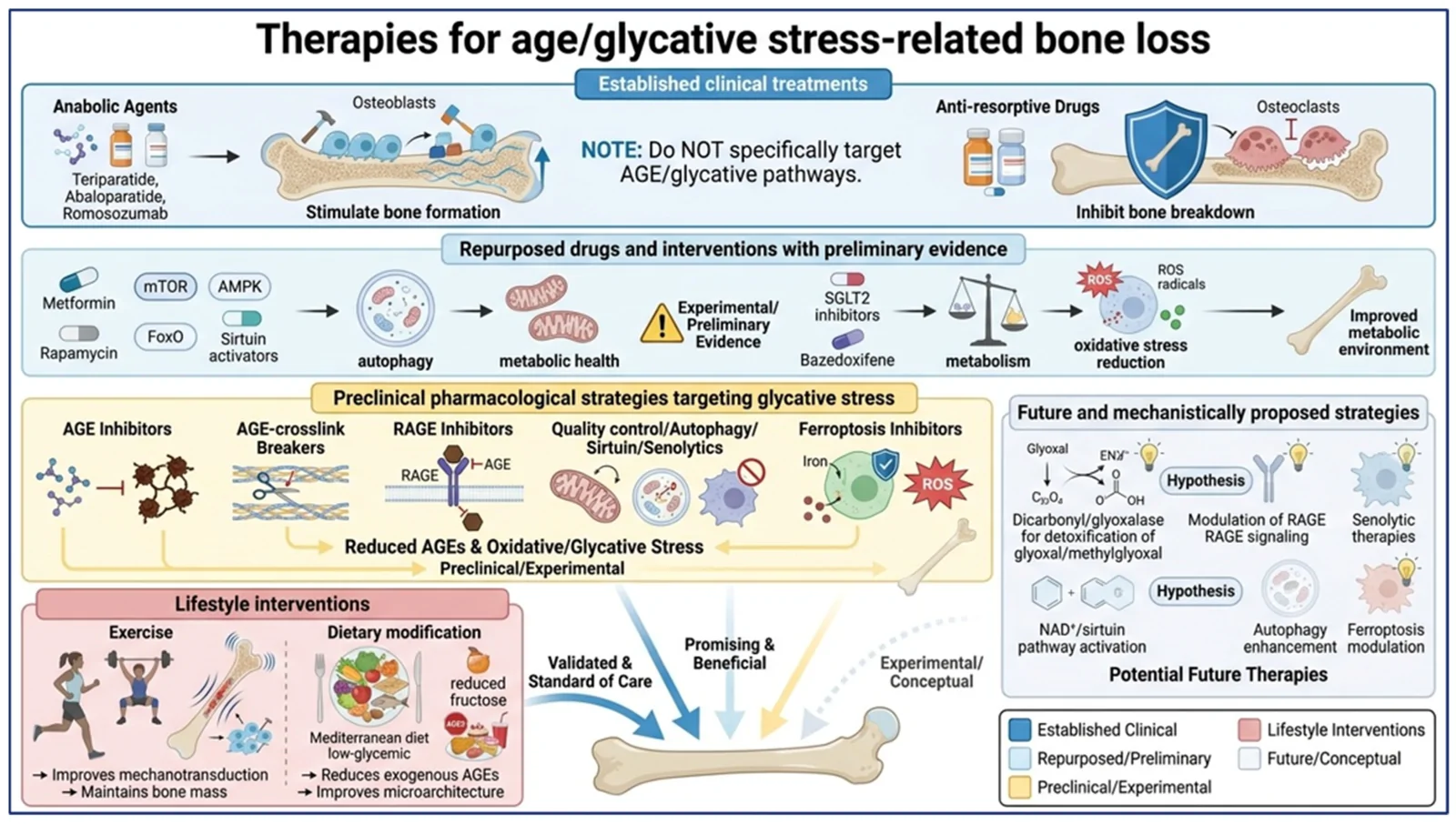
Current, repurposed, preclinical and future therapeutic strategies targeting age- and glycative stress-related bone loss. Tiered overview of therapeutic approaches, color-coded by level of clinical validation and converging on a central bone icon via arrows ranging from validated standard of care to experimental/conceptual. **Top row** (“Established clinical treatments”): Anabolic agents (teriparatide, abaloparatide, romosozumab) stimulate osteoblast-mediated bone formation, and anti-resorptive drugs inhibit osteoclast-mediated bone breakdown; a note specifies that neither class specifically targets AGE/glycative pathways. **Second row** (“Repurposed drugs and interventions with preliminary evidence”): metformin, rapamycin and mTOR/AMPK/FoxO/sirtuin-activator compounds promote autophagy and metabolic health, while SGLT2 inhibitors and bazedoxifene improve metabolism and reduce oxidative stress (ROS radicals), together yielding an improved metabolic environment. **Third row** (“Preclinical pharmacological strategies targeting glycative stress”): AGE inhibitors, AGE-crosslink breakers, RAGE inhibitors, quality-control/autophagy/sirtuin/senolytic agents and ferroptosis inhibitors converge to reduce AGEs and oxidative/glycative stress. **Bottom left** (“Lifestyle interventions”): Exercise improves mechanotransduction and maintains bone mass, while dietary modification (Mediterranean/low-glycemic-index diet, reduced fructose intake) reduces exogenous AGE exposure and improves bone microarchitecture. **Right panel** (“Future and mechanistically proposed strategies,” marked as hypotheses): Dicarbonyl/glyoxalase-mediated detoxification of glyoxal/methylglyoxal, modulation of RAGE signaling, senolytic therapies, NAD^+^/sirtuin pathway activation, autophagy enhancement and ferroptosis modulation are proposed as potential future therapies. The bottom legend maps the color scheme (established clinical, repurposed/preliminary, preclinical/experimental, lifestyle interventions, future/conceptual) to the corresponding arrows converging on the bone diagram, spanning from validated and standard-of-care interventions through promising/beneficial approaches to experimental/conceptual strategies.

Teriparatide, a synthetic analogue comprising the first 34 amino acids of PTH, and abaloparatide, a structural analogue of PTHrP, are PTH1R agonists that stimulate bone formation by enhancing osteoblast activity and differentiation and by reducing osteoblast apoptosis [116]. Teriparatide may additionally activate Wnt signaling and has been associated with improved DNA repair and reduced expression of senescence-associated markers such as p16^Ink4a [117]. Romosozumab, a monoclonal antibody targeting sclerostin, promotes bone formation by relieving inhibition of Wnt and BMP signaling while also reducing bone resorption. Clinical studies have demonstrated substantial increases in BMD and bone strength following romosozumab treatment [118]. However, its anabolic effect decreases after treatment discontinuation, and subsequent anti-resorptive therapy is generally required to maintain the skeletal benefits (Figure 8).

Anti-resorptive therapies, including bisphosphonates and denosumab, remain a cornerstone of osteoporosis management. Bisphosphonates such as alendronate, risedronate, and zoledronate inhibit osteoclast function by targeting farnesyl diphosphate synthase, whereas denosumab prevents RANKL–RANK signaling and osteoclast differentiation [119]. Although these therapies do not specifically target glycative stress, they provide clinically established mechanisms for reducing skeletal fragility associated with increased bone resorption (Figure 8).

Importantly, these treatments should not be interpreted as therapies specifically targeting AGE accumulation or glycative damage. Rather, they represent established approaches for osteoporosis that may counteract some of the downstream consequences of skeletal aging.

### 7.2. Repurposed Drugs and Interventions with Preliminary Evidence

Several clinically available drugs and pharmacological agents have been proposed to influence pathways implicated in glycative and age-related skeletal deterioration, although their specific efficacy against glycative bone disease remains insufficiently established.

Metformin, rapamycin and mTOR-modulating compounds, as well as AMPK-activating strategies such as AICAR, have been investigated for their ability to restore autophagic and metabolic homeostasis in bone cells. Similarly, compounds targeting the AMPK/FoxO and sirtuin pathways, including metformin and certain metabolic modulators, have shown protective effects against oxidative stress, senescence and impaired osteogenesis in experimental models [56,108]. However, these findings should be considered preliminary and cannot currently be interpreted as evidence that these agents treat glycative bone disease in humans (Figure 8).

Other pharmacological approaches may indirectly influence glycative damage. For example, SGLT2 inhibitors may modify metabolic and glycaemic parameters and have been proposed to influence AGE-related pathways, although their specific effects on glycative bone damage remain incompletely characterized. Likewise, bazedoxifene has been reported to modulate oxidative stress and the lysyl oxidase/pentosidine balance in experimental models [120]. These observations provide a rationale for further investigation but do not establish these drugs as antiglycative treatments (Figure 8).

### 7.3. Preclinical Pharmacological Strategies Targeting Glycative Stress

More direct approaches targeting AGE formation, AGE crosslinking, or AGE–RAGE signaling remain predominantly preclinical. Classical AGE inhibitors, including aminoguanidine and pyridoxamine, and AGE crosslink breakers such as alagebrium (ALT-711), have shown beneficial effects in experimental models by reducing AGE accumulation or crosslinking and improving tissue mechanical properties. However, their clinical development has been limited by toxicity, bioavailability, or insufficient efficacy [14,82,121]. Emerging strategies include dicarbonyl scavengers, glyoxalase-mimetic systems, and compounds designed to enhance endogenous detoxification pathways, but these approaches require further validation before clinical translation [15,18] (Figure 8).

RAGE signaling represents another potential therapeutic target. Small-molecule inhibitors such as FPS-ZM1 and other RAGE-directed compounds, as well as soluble RAGE-based approaches, have shown protective effects in experimental models by reducing RAGE-dependent oxidative and inflammatory signaling. Nevertheless, these strategies are still considered experimental and are not established treatments for glycative bone disease. Similarly, compounds such as FPS-ZM1 should currently be considered experimental tools for investigating the AGE–RAGE axis rather than clinically validated therapeutic agents [26] (Figure 8).

Modulation of cellular quality-control pathways represents another emerging strategy. Experimental studies suggest that activation of AMPK, restoration of autophagic and mitophagic flux, and enhancement of sirtuin-dependent mitochondrial protection may attenuate AGE-induced cellular dysfunction [56,109]. Likewise, senolytic and senomorphic strategies, including dasatinib/quercetin, fisetin, navitoclax, JAK inhibitors, and mTOR inhibitors, have shown beneficial effects on skeletal aging in experimental models by reducing senescent cell burden or suppressing the SASP [50,122,123]. However, these compounds remain investigational in the context of skeletal aging and should not be considered established treatments (Figure 8).

Ferroptosis represents a further potential therapeutic target. Experimental studies indicate that ferroptosis inhibitors, iron-modulating approaches, and strategies aimed at preserving the NRF2–GPX4–SLC7A11 antioxidant system may protect osteoblasts and osteocytes from metabolic and glycative damage. Importantly, compounds such as ferrostatin-1 and liproxstatin-1 remain preclinical research tools and are not established therapies for glycative bone disease [124]. Further studies are required to determine whether targeting ferroptosis can produce meaningful skeletal benefits in vivo (Figure 8).

### 7.4. Lifestyle Interventions

Lifestyle interventions represent the most clinically accessible strategies for modifying several upstream factors associated with both skeletal aging and glycative stress. Regular physical activity promotes bone mechanotransduction, improves metabolic health, and may reduce systemic oxidative and inflammatory stress. Experimental evidence also suggests that exercise can reduce AGE accumulation and oxidative damage while preserving trabecular and cortical bone microarchitecture [125].

Beyond endogenous formation, dietary intake represents an additional source of systemic AGE exposure. Foods subjected to dry-heat processing, particularly meat and other protein-rich foods, can contain substantial amounts of dietary AGEs, with cooking conditions strongly influencing their formation [126,127]. Meat has been reported among the food groups with particularly high AGE content, although dietary AGE content varies substantially according to food type and cooking method [127]. Observational evidence has also linked dietary patterns with metabolic disease risk, with diets characterized by greater consumption of meat and other foods being associated with adverse metabolic profiles, including higher diabetes risk [128]. Saturated-fat-rich diets may further contribute to metabolic disturbances, including altered postprandial glycaemia and hepatic lipid accumulation, although these findings do not establish a direct relationship between saturated fat intake and AGE accumulation in bone [129]. Besides diet, tobacco and, in CKD, the failure of renal clearance further amplify the systemic AGE burden, which also includes uraemic toxins such as indoxyl sulfate (IS) and p-cresyl sulfate (pCS) [19] (Figure 8).

Dietary strategies may similarly influence glycative burden. Mediterranean-style and low-glycaemic-index diets may reduce endogenous AGE formation, while reducing consumption of foods rich in exogenous AGEs may decrease dietary AGE exposure. These approaches have also been associated with favorable effects on metabolic health, inflammation, and potentially glyoxalase and RAGE-related pathways [14,130]. Nevertheless, the specific contribution of dietary AGE restriction to the prevention of skeletal fragility requires further clinical investigation (Figure 8).

### 7.5. Future and Mechanistically Proposed Strategies

Beyond currently available interventions, several approaches have been proposed on the basis of mechanistic evidence. These include enhancing endogenous dicarbonyl detoxification through glyoxalase pathways, modulating RAGE signaling, restoring NAD^+^/sirtuin activity, targeting senescent cells, enhancing autophagy and mitophagy, preventing ferroptosis, and restoring defective mechanotransduction.

Although these mechanisms provide attractive therapeutic targets, their potential relevance should not be equated with clinical efficacy. In particular, ferrostatin-1, liproxstatin-1, FPS-ZM1, several senolytic agents, and experimental autophagy modulators remain at the preclinical or mechanistic stage and require validation in appropriate animal models and, ultimately, clinical studies (Figure 8).

Overall, current clinical management of skeletal fragility remains based primarily on established anabolic and anti-resorptive therapies, together with lifestyle interventions. In contrast, therapies specifically targeting glycative stress and its downstream consequences remain experimental. Moreover, modulation of these pathways by the proposed therapies may be associated with unintended off-target effects, safety concerns, and interference with physiological processes essential for tissue homeostasis and repair. Consequently, these approaches should be interpreted with caution, and their therapeutic potential must be carefully balanced against potential risks before clinical translation. Nevertheless, given the interconnected nature of AGE accumulation, oxidative stress, inflammation, senescence, mitochondrial dysfunction, and impaired mechanotransduction, simultaneous modulation of several of these pathways may represent a promising future therapeutic concept. However, whether such a multilevel strategy provides superior clinical benefit compared with current standard treatments remains speculative and requires direct experimental and clinical validation.

## 8. Conclusions

Aging and glycative stress are major contributors to skeletal fragility that share several pathogenic mechanisms, including oxidative stress, chronic inflammation, mitochondrial dysfunction, cellular senescence, impaired autophagy, ferroptosis, altered RANKL/OPG signaling, and defective osteocyte mechanotransduction. These convergent pathways impair bone remodeling and increase fracture risk.

Despite these similarities, important differences exist. Aging is primarily associated with stem-cell exhaustion, hormonal changes, osteocyte network degeneration, and progressive loss of bone mass and microarchitecture. In contrast, glycative stress is driven by AGE accumulation and AGE–RAGE signaling, leading predominantly to impaired bone quality, altered collagen properties, and defective mechanosensing despite often preserved bone mineral density.

Although several mechanisms have emerged as promising therapeutic targets, important questions remain regarding the roles of AGE-mediated osteoclast regulation, Piezo1, YAP/TAZ, NLRP3 signaling, and the relative contributions of matrix- versus cell-mediated dysfunction. An integrated mechanobiological view of aging and glycative stress may facilitate the identification of both shared and condition-specific therapeutic targets for skeletal fragility.

## 9. Future Perspectives

Future research should focus on validating the proposed mechanisms in physiologically relevant in vivo and human models, with particular attention to osteocyte mechanotransduction, AGE-induced matrix alterations, senescence, autophagy dysfunction, and ferroptosis. Comparative studies across aging, type 2 diabetes, chronic kidney disease, and dietary AGE exposure will be essential to distinguish shared from context-specific mechanisms.

From a translational perspective, anabolic and anti-resorptive therapies remain the clinical standard, whereas interventions targeting AGE accumulation, RAGE signaling, senescence, autophagy, mitochondrial dysfunction, NAD^+^ metabolism, and ferroptosis should currently be regarded as experimental. Future therapeutic strategies will likely require combined approaches addressing glycative damage, inflammation, impaired mechanotransduction, and age-related cellular dysfunction. However, careful evaluation of long-term safety, specificity, and clinical efficacy will be essential before these approaches can be translated into routine clinical practice.

## Data Availability

No new data were created or analyzed in this study. Data sharing is not applicable to this article.

## Abbreviations

AGE/AGEs	Advanced glycation end-product(s)
AGER	Advanced glycation end-product-specific receptor
AGOE/AGOEs	Advanced glycoxidation end-product(s)
AICAR	5-Aminoimidazole-4-carboxamide ribonucleotide
Akt	Protein kinase B
ALP	Alkaline phosphatase
ALT-711	Alagebrium chloride
AMPK	AMP-activated protein kinase
ASC	Apoptosis-associated speck-like protein containing a CARD
ATF3	Activating transcription factor 3
ATF4	Activating transcription factor 4
ATP	Adenosine triphosphate
Bax	Bcl-2-associated X protein
Bcl-2	B-cell lymphoma 2
BMD	Bone mineral density
BMP	Bone morphogenetic protein
BMSC(s)	Bone marrow (mesenchymal) stromal/stem cell(s)
CHOP	C/EBP homologous protein
CSF-1	Colony-stimulating factor 1
Cx43	Connexin 43
DAMPs	Damage-associated molecular patterns
DC-STAMP	Dendritic cell-specific transmembrane protein
DJ-1/Park7	Parkinson disease protein 7
Dkk1	Dickkopf-related protein 1
ERα	Estrogen receptor alpha
ERK/ERK1/2	Extracellular signal-regulated kinase (1/2)
FGFR1/FGFR2	Fibroblast growth factor receptor 1/2
FOXO/FOXO3	Forkhead box O
FPS-ZM1	Small-molecule RAGE antagonist
FtMt	Mitochondrial ferritin
GM-CSF	Granulocyte-macrophage colony-stimulating factor
GPCR	G protein-coupled receptor
GPX4	Glutathione peroxidase 4
HMGB1	High mobility group box 1
ICAM-1	Intercellular adhesion molecule 1
IGF-1	Insulin-like growth factor 1
IL-1β	Interleukin-1 beta
IL-6	Interleukin-6
IL-11	Interleukin-11
IL-18	Interleukin-18
JAK	Janus kinase
JNK	c-Jun N-terminal kinase
LCN	Lacunocanalicular network
LFA-1	Lymphocyte function-associated antigen 1
MAPK/p38 MAPK	Mitogen-activated protein kinase (p38 isoform)
MCP-1	Monocyte chemoattractant protein 1
MEK	Mitogen-activated protein kinase kinase
MGO	Methylglyoxal
MIP-1α/β	Macrophage inflammatory protein 1 alpha/beta
MMP(s)	Matrix metalloproteinase(s)
mTOR	Mechanistic target of rapamycin
NAD+	Nicotinamide adenine dinucleotide
NFATc1	Nuclear factor of activated T cells, cytoplasmic 1
NF-κB	Nuclear factor kappa-light-chain-enhancer of activated B cells
NLRP3	NOD-, LRR- and pyrin domain-containing protein 3
NRF2	Nuclear factor erythroid 2-related factor 2
OPA1	Optic atrophy 1
OPG	Osteoprotegerin
OXPHOS	Oxidative phosphorylation
P2X7	P2X purinoceptor 7
p16INK4a	Cyclin-dependent kinase inhibitor 2A
p21Cip1	Cyclin-dependent kinase inhibitor 1A
PI3K	Phosphoinositide 3-kinase
PTEN	Phosphatase and tensin homolog
PTH1R	Parathyroid hormone 1 receptor
PTHrP	Parathyroid hormone-related protein
PUFA	Polyunsaturated fatty acid
Rac1	Ras-related C3 botulinum toxin substrate 1
RAGE	Receptor for advanced glycation end-products
RANK	Receptor activator of nuclear factor kappa-B
RANKL	Receptor activator of nuclear factor kappa-B ligand
RANTES	Regulated on activation, normal T cell expressed and secreted (CCL5)
RCS	Reactive carbonyl species
ROS	Reactive oxygen species
RUNX2	Runt-related transcription factor 2
SASP	Senescence-associated secretory phenotype
SGLT2	Sodium-glucose cotransporter 2
Shh	Sonic hedgehog
SIRT1	Sirtuin 1
SLC7A11	Solute carrier family 7 member 11
Smad	Small mothers against decapentaplegic
SOST	Sclerostin
SP7 (Osterix)	Sp7 transcription factor
SSPCs	Skeletal stem/progenitor cells
STAT3	Signal transducer and activator of transcription 3
T2DM	Type 2 diabetes mellitus
TAZ	Transcriptional coactivator with PDZ-binding motif (WWTR1)
TfR1	Transferrin receptor 1
TGF-β	Transforming growth factor beta
TLR4	Toll-like receptor 4
TNF-α	Tumor necrosis factor alpha
TRAP	Tartrate-resistant acid phosphatase
ULK1	Unc-51-like autophagy-activating kinase 1
UPS	Ubiquitin-proteasome system
VDR	Vitamin D receptor
VEGF	Vascular endothelial growth factor
Wnt	Wingless/Integrated
YAP	Yes-associated protein
